# Geohazard assessment of Mexico City’s Metro system derived from SAR interferometry observations

**DOI:** 10.1038/s41598-024-53525-y

**Published:** 2024-03-12

**Authors:** Darío Solano-Rojas, Shimon Wdowinski, Enrique Cabral-Cano, Batuhan Osmanoğlu

**Affiliations:** 1 División de Ingeniería en Ciencias de la Tierra, Facultad de Ingeniería, Universidad Nacional Autónoma de México, 04360 Mexico City, CDMX Mexico; 2 Department of Earth and Environment, Institute of Environment, Florida International University, Miami, FL 33199 USA; 3 School of Marine and Atmospheric Science, University of Miami, 4600 Rickenbacker Causeway, Miami, FL 33149-1098 USA; 4 Departamento de Geomagnetismo y Exploración, Instituto de Geofísica, Universidad Nacional Autónoma de México, Ciudad Universitaria, 04510 Mexico City, CDMX Mexico; 5 NASA Goddard Space Flight Center, Greenbelt, MD 20771 USA

**Keywords:** Civil engineering, Natural hazards

## Abstract

Land subsidence rates in Mexico City reach 500 mm/year, causing progressive damage to the city’s core infrastructure, including the Metro system. A deadly overpass collapse in 2021, along a Metro line that had operated for less than 10 years, brought subsidence-related structural damage to the attention of the system’s authorities and led to major repairs to two of the twelve Metro lines. Still, the need for quantifying the magnitude and extent of subsidence affecting the Metro system’s widespread infrastructure prevails. Using a wealth of satellite radar interferometry observations, levelling surveys, subsurface profiles, linear gradient and differential displacement analyses, and structural-engineering parameters, we assess the vulnerability of the Metro system’s street-level and elevated segments to land subsidence. Our results reveal that high subsidence velocity gradients occur over sharp transitional zones between stable and fast-subsiding areas, reaching values of 1 $\times \,10 ^{-3}$ year^-1^, resulting in slope changes up to 3.5% over a 20-year period and differential displacements between columns. Our findings suggest locations where the consequences of subsidence have compromised the train’s braking safety design, increased railway flooding hazard, produced railway bending, and reduced the conceived 50-year service life of the Metro’s elevated overpasses.

## Introduction

**Figure 1:**
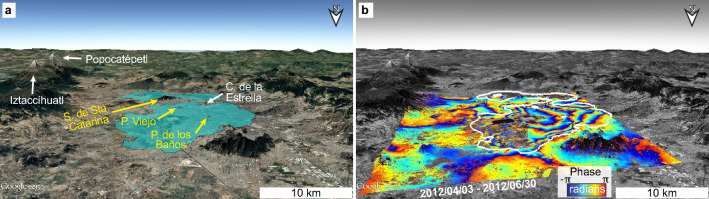
Perspective view of the Mexico City basin overlain a GoogleEarth image. (**a**) The blue polygon marks the location of the former lake area according to the geotechnical lake zone^7^. Yellow labels indicate locations mentioned in the text and white labels name topographic highs for reference. (**b**) An 88-day COSMO-SkyMed interferogram showing phase changes indicative of land subsidence. Each cycle from -$\pi$ to $\pi$ represents 15.5 mm of displacement in the satellite’s line of sight. The white contour marks the boundary of the lake zone. Notice that subsidence occurs mostly within the lake zone. Maps created using Google Earth Pro 7.3 (https://www.google.com/intl/en/earth). Satellite imagery credits: Landsat/Copernicus.

**Figure 2:**
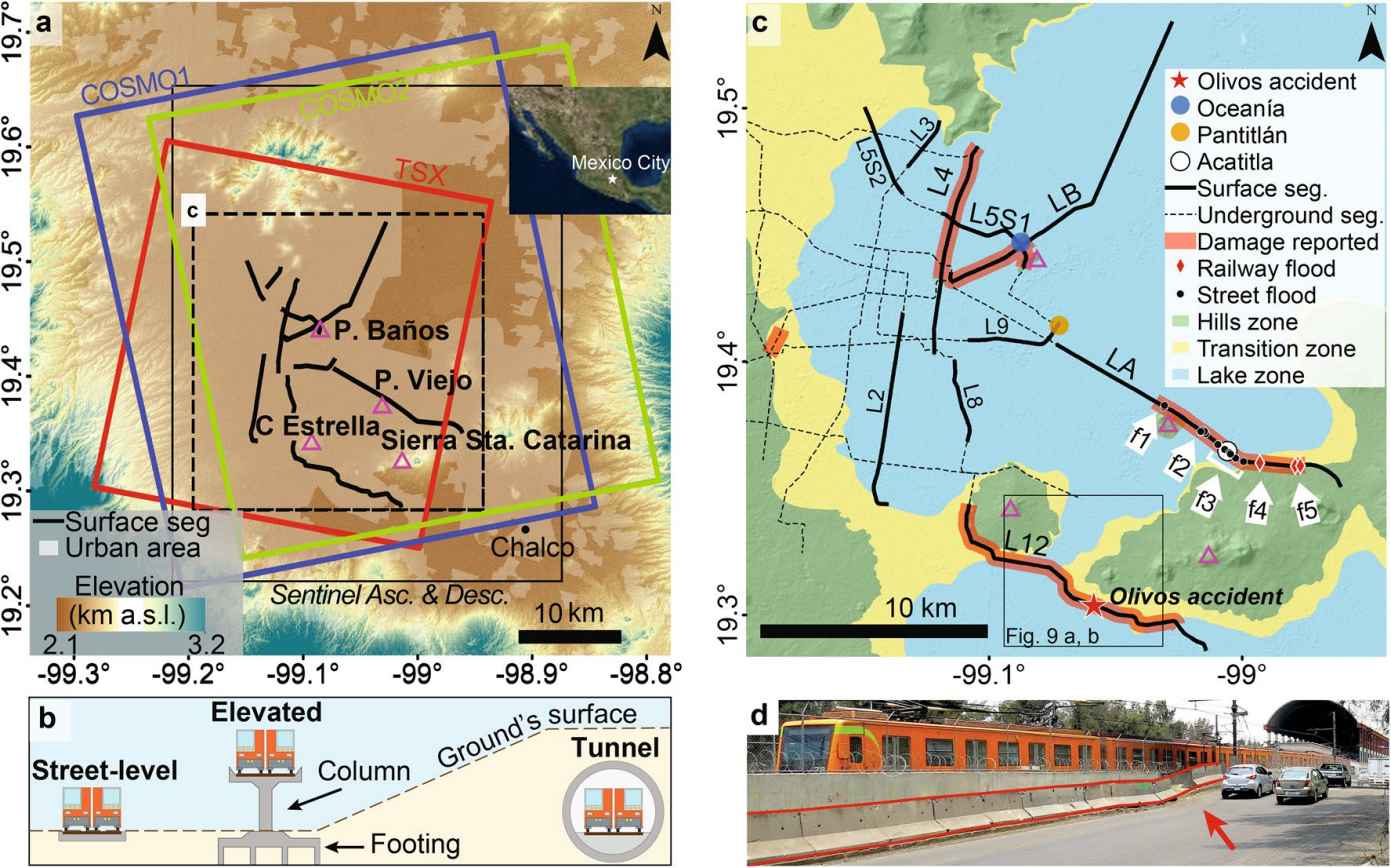
SAR datasets used in this study, railway engineering designs used by the Metro and locations of subsidence-related damage reports. (**a**) SRTM elevation map overlayed by the footprints of the five SAR datasets described in the Results section. Red, blue and green polygons correspond to the three X-band datasets, here named TSX, COSMO1, and COSMO2, whereas the solid black polygon corresponds to the two C-band datasets from Sentinel in both Ascending and Descending orbits. Insert shows the location of Mexico City. Solid black lines indicate the Metro’s surface segments and the dashed black frame indicates the coverage of (**c**). Pink triangles mark local topographic highs. (**b**) Illustration of street-level, elevated, and underground Metro line designs used in Mexico City. (**c**) Damaged segments and stations of the Metro system (as described in Supplementary Table S1 online) overlaying the city’s official geotechnical zoning^7^. White arrows indicate clusters of localized street-flood and railway-flood reports along LA associated with train shutdowns^75,76^ (Supplementary Text S1). (**d**) Differential subsidence at Acatitla Station (location in (**c**)), as indicated by the tilted road traffic barriers (marked by two red lines). ArcMap 10.2 (https://www.esri.com/software/arcgis) was used to produce the maps, which show elevation and shaded relief from SRTM data (https://earthexplorer.usgs.gov).

Mexico City’s Metro system transports more than 4 million passengers per day^1^; hence, any impact on the system’s performance has serious transit-time and financial consequences to its passengers^2^ and, ultimately, to the whole city’s productivity. Land subsidence in Mexico City is one of the fastest in the world^3^, reaching up to 500 mm/year^4^, and occurring mainly as the response to the aggressive groundwater extraction over the past century from the highly-heterogeneous compressible stratigraphic sequence beneath the city^5,6^. Highest subsidence rates occur within the former lake area^7^ (Fig. 1a, 1b) in correlation with the thickness of the uppermost clay-rich lithological unit^4,8^, whereas non-subsiding areas correspond to volcanic mountains around and within the lacustrine zone (Figs. 1a, 2c). The subsidence occurs deferentially, particularly along transitional zones between stable volcanic rocks (hill zone; green area in Fig. 2c) and highly compressible sediments (lake zone, blue area in Fig. 2c)^6^. Notably, differential subsidence has compromised some of the Metro’s structural components, which has led to the system malfunctioning (see listing of subsidence-related malfunctioning in Supplementary Table S1 online, Supplementary Text S1 online).

Locating subsidence-related problems in the Metro’s infrastructure is a challenging task because they vary according to the railway’s engineering design: street-level, elevated, or underground (Fig. 2b). Openly-available official reports are scarce and provide limited details on damage occurrence and location^9,10^; media reports provide additional information on such aspects (Supplementary Text S1 online, Supplementary Table S1 online). Reports indicate widespread damage throughout the system, but with increased occurrence along LA, LB, L4, L5S1, and L12, which correspond to surface segments (street-level and elevated) (Fig. 2a, c). Damage evidence includes cracks, faults, and structural collapses over a variety of supporting structures, as well as railway deformation and topographic slope changes (e.g. Fig. 2d). Such damage has led to recurrent flooding along surface railways as well as neighbouring streets after heavy rains, and malfunctions including reduced train speed, performance decline, service interruptions, and accidents.

An accident in particular, which resulted in more than 20 casualties, raised concerns about the current state of the Metro system’s infrastructure, even though a potential contribution of subsidence-related damage to the accident’s occurrence has not yet been clearly established. On May 3rd, 2021, an overpass located close to Olivos station collapsed onto the road as a train was travelling over it^11,12^ (see location in Fig. 2c). Post-collapse analyses detected design and construction deficiencies that contributed to the collapse and suggested that differential displacements between the columns supporting the overpass could have also been a contributing factor^13,14^. Post-collapse inspections of elevated and underground structures and railways along L12 revealed structural damage that led to major spending on repairs in various sectors of the system^11,15^. However, the lack of geodetic instrumentation for permanently monitoring displacements and the scarcity of pre-collapse levelling surveys along the structure impeded determining quantitatively whether or not subsidence could be linked to the occurrence of the accident^13,14^.

Mexico City subsidence has been studied using traditional (i.e. levelling) and advanced geodetic techniques such as satellite Interferometric SAR (InSAR) and GPS (e.g.^4,6,8,16,17^), which detected highest subsidence rates eastward from Peñón de los Baños and in Chalco (locations in Fig. 2a). GPS and InSAR time series analyses^4,16,17^, as well as the systematic comparison of InSAR velocity maps^4^, revealed high linearity, no significant annual variations, and a dominant vertical component of the subsidence process in Mexico City. Post-processing techniques applied to InSAR velocity maps, such as subsidence gradient^6^, background value removal^16^, angular distortion^17,18^ and band-pass filtering^19^ have been used to quantify differential subsidence in Mexico City.

Only two studies, heretofore, have linked InSAR-measured subsidence variations with damage to the Metro’s L4, LB, and Pantitlán station^16,19^. However, those studies have focused on quantifying differential displacements only over localized areas. Therefore, the need for an in-depth study of the geohazard to the Metro’s infrastructure has prevailed. Here, we use a wealth of C- and X-band satellite SAR data and multi-temporal InSAR techniques to calculate subsidence velocities along the Metro system’s infrastructure and quantify differential subsidence. We then use structural engineering parameters and a compilation of subsidence-related damage reports to determine segments where deformation is likely to adversely affect the design of the system according to construction codes and the railway’s intended design. In particular, we apply our approach to analyze the possible contribution of differential subsidence to the May 3rd, 2021 Olivos viaduct collapse (see location in Fig. 2c).

## Results

In this section, we present the InSAR results we obtain from C- and X-band datasets. Details on the methodology selection criteria used to process each dataset are provided in the “Materials and methods” section. Here, we first describe and compare vertical velocities obtained from InSAR over Mexico City and identify the sectors where our results differ the most. Because the main objective of this investigation is to assess the geohazard to the Metro system, we then proceed to inspect and compare calculated vertical velocities and gradients along the Metro lines. Additionally, we illustrate that unwrapping errors contribute significantly to observed differences among velocity results.

### InSAR results

**Figure 3:**
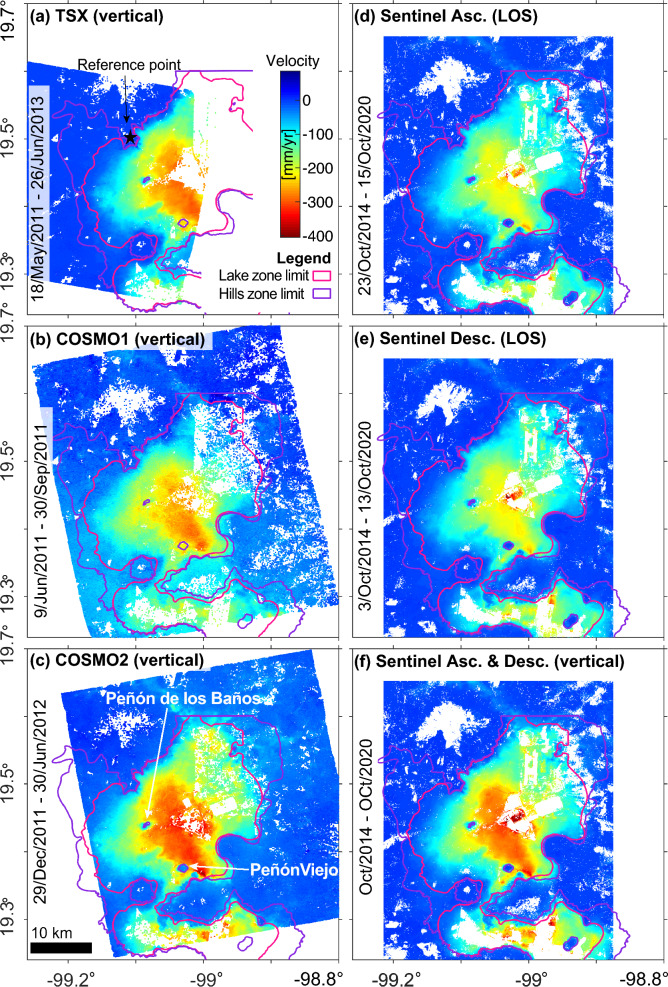
Velocity maps derived from the five datasets used for this study. (**a**) TSX, (**b**) COSMO1, and (**c**) COSMO2 vertical velocity results. The purple and pink lines mark the geotechnical classification outlines (Fig. 2c). The black star in (**a**) marks the location of the reference point common to all datasets processed in this study. (**d,e**) Sentinel-1A, B results from ascending and descending orbits, respectively, in LOS observation geometry. (**f**) Sentinel vertical velocities obtained after combining both ascending and descending orbits. Maps created using Matlab R2015b (https://www.mathworks.com).

Our results were based on three X-band datasets processed at full resolution using a Persistent Scatterers (PS) Interferometry (PSI) technique and two C-band datasets processed using multilooking and the Small Baselines (SBAS) approach (see “Materials and methods”). The X-band datasets (spatial resolution of 3 m) were acquired by the TerraSAR-X and COSMO-SkyMed satellites from May 2011 to June 2013. Such X-band datasets were divided according to sensor’s type, acquisition orbits and named TSX, COSMO1, and COSMO2 (Fig. 2a, Supplementary Fig. S1 online, Supplementary Tables S2 and S3 online). Each X-band dataset was processed to calculate velocity maps in Line of Sight (LOS) geometry, from which vertical velocities were estimated (Fig. 3a–c). The C-band datasets (spatial resolution of $\sim$15 m) were acquired by the Sentinel-A and B satellites in ascending and descending modes from October 2014 to October 2020 (Supplementary Table S3 online). A LOS velocity map was obtained from each C-band dataset (Fig. 3d, e), to later combine both C-band velocity results and calculate the vertical component (Fig. 3f). In total, we obtain four vertical velocity maps (Fig. 3a–c, f). Spatial patterns and rates in our results compare very well with those reported in previous studies^4,6,16,17^. In all cases, the fastest subsiding zones are mostly constrained within the outlines of the lake zone and smoothly transition outwards to stable areas (Fig. 3a–f). However, subsidence velocities differ the most over isolated volcanic areas embedded in deformable lacustrine deposits. For example, the volcanic rocks of Peñón de los Baños and Peñón Viejo (see location in Figs. 2a, 3c) are stable areas, as determined by levelling surveys^20,21^. The results from TSX and COSMO1 analyses indicate that such two areas deform (Fig. 3a, b). In contrast, the results from COSMO2 and Sentinel-1 datasets (Fig. 3c–f) do display such two areas as stable, in agreement with two other recent studies^4,17^.

### InSAR results comparison

**Figure 4:**
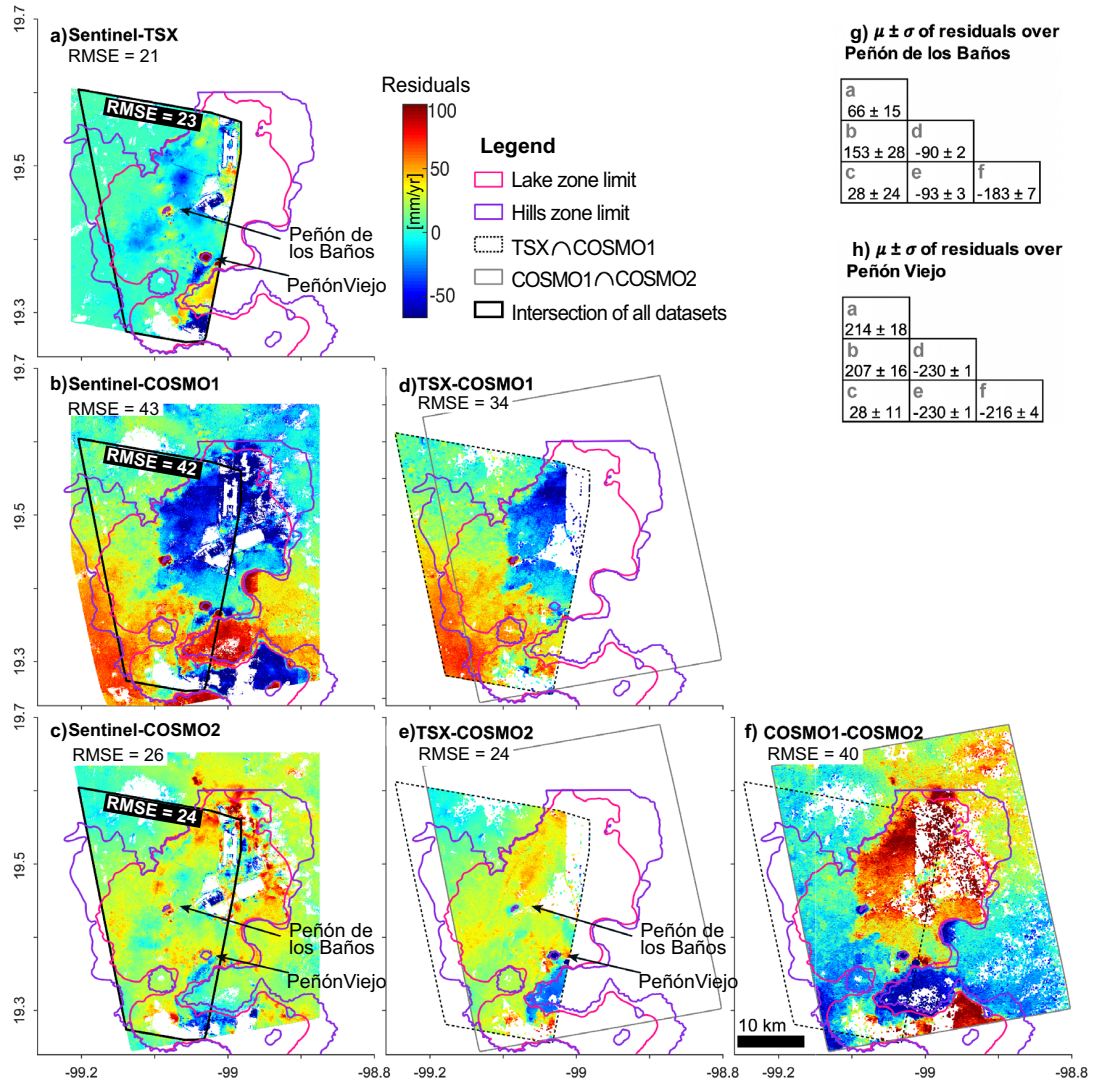
Maps with residual vertical velocities from all datasets and their corresponding RMSE. (**a–c**) Sentinel vertical velocities (Fig. 2f) minus TSX, COSMO1, and COSMO2 results (Fig. 2a–c), respectively. (**a–c**) Include a black polygon indicating the overlapping area of all datasets and an additional label indicating the RMSE calculated within such polygon. (**d**) TSX minus COSMO1, (**e**) TSX minus COSMO2, (**f**) COSMO1 minus COSMO2. In (**d–f**), the dashed back polygon is the common area between TSX and COSMO1 datasets, and the grey polygon is the common area between COSMO1 and COSMO2. Pink and purple lines in all frames correspond to the lake and hills geotechnical zone boundaries shown in Fig. 2c and Supplementary Fig. S2 online. (**g,h**) Calculated mean ($\mu$) and standard deviation ($\sigma$) of the residuals in (**a–f**) over Peñón de los Baños and Peñon Viejo. Units are mm/year. Maps created using Matlab R2015b (https://www.mathworks.com).

In order to compare the four vertical velocity maps we obtained, we calculate the difference of the velocity estimations, or residual velocities, and present them in Fig. 4a–f. We additionally calculate the root-mean-square error (RMSE) for each map (white labels in Fig. 4a–f). The two C-band LOS velocity estimations in this study rely on $\sim$10x as many scenes as compared to X-band datasets (see Supplementary Table S3 online, see “Materials and methods”). In general, the greater number of scenes available for the velocity calculation yields smaller associated uncertainties (see Supplementary Fig. S8 online). The velocity estimations from 6-year-long Sentinel results we produce yield uncertainties up to one order of magnitude smaller than the results produced by the X-band datasets (see Supplementary Fig. S9 online). Thus, we use vertical velocities from Sentinel to compare and validate the subsidence patterns obtained from the higher-spatial-resolution X-band datasets. The comparison we perform reveals a wide range of residuals roughly limited by the geotechnical boundaries of the lake zone and over stable areas embedded in deformable sediments (e.g. Fig. 4b). The residuals map between Sentinel and COSMO1 (Fig. 4b) shows the largest residuals of all (RMSE = 43). The residuals between Sentinel and COSMO2 (RMSE = 26) show the largest values in the northeastern and southern portions of the lake area (Fig. 4c). The residuals map between Sentinel and TSX (Fig. 4a) shows the greatest values in the southern sector, and produces, apparently, the smallest RMSE among all X-band datasets^22^. However, RMSE values calculated within the area common to all four datasets (black polygon and black labels in Fig. 4a–c show that, when compared to the Sentinel estimates, TSX and COSMO2 results provide practically the same RMSE (23 vs 24). The residuals maps among X-band datasets show that COSMO1 (Fig. 4d, f) produces higher residuals when compared to any other dataset (RMSE > 34), while the residuals between TSX and COSMO2 results (Fig. 4e) show that they are very similar to each other (RMSE = 24). Penón de los Baños and Peñón Viejo are particularly relevant to this study, and thus, we analyze even further the agreement of our results over those areas by calculating the mean and standard deviation of the residuals (Fig. 4g, h). Average residuals range from 28 to 230 mm/year over Peñón de los Baños y Peñón Viejo (Fig. 4g, h). Notably, the residuals produced between Sentinel and COSMO2 results are the smallest among all at 28 ± 24 mm/year and 28 ± 11 mm/year over Penón de los Baños and Peñón Viejo, respectively (Fig. 4g, h).

### Vertical velocities and gradients along Metro lines

Mexico City Metro System’s narrow infrastructure features span all over the city and, thus, are subject to a wide range of subsidence velocities. Therefore, adequate sampling density and spatial coverage of subsidence velocities are essential. The final sampling in space available from each dataset varies depending on the spatial resolution of the dataset and the multi-looking used during the implementation of a given InSAR algorithm. In our case, COSMO1, COSMO2, and TSX results provide denser sampling in space (spaced as closely as 3 m) than Sentinel results (spaced as closely as 30 m). In terms of coverage, TSX and COSMO1 datasets do have limitations towards the eastern portion of the city, while COSMO2 results cover entirely the Metro’s surface segments (compare Supplementary Fig. S2a, S2b versus S2c online).

We obtain average velocities within 30x10m rectangles along surface metro lines from COSMO1, COSMO2, and TSX results, assign the nearest value to each rectangle from Sentinel results and present them in Supplementary Fig. S3 online (see “Materials and methods”). Overall, velocities along the Metro’s surface railways vary between 0 and $-300$ mm/year and are consistent with all the solutions (Supplementary Fig. S3 online). However, we observe important differences in TSX and COSMO1 with respect to COSMO2 and Sentinel results. For example, we identify from Supplementary Fig. S3 online that Sentinel and COSMO2 velocities increase and/or decrease consistently along km 4 to 5 of L5S1, km 5.5 to 13 of LA, and km 6 to 14 of L12, while COSMO1 and TSX solutions show flatter profiles. Such misfit between COSMO1 and TXS with respect to Sentinel results is consistent with the residuals analysis from our InSAR results comparison.

We calculate velocity gradients from the average velocities for estimating differential displacements along the Metro lines from COSMO2 results and compare them against gradients calculated from Sentinel results (see Supplementary Figs. S3 and S4 online). Velocity gradients calculated from Sentinel and COSMO2 results vary mostly in the interval ± 0.5 $\times$ 10^-3^ year ^-1^ (Supplementary Fig. S4 online). However, gradients calculated from Sentinel results produce a smoother curve along all metro lines (Supplementary Fig. S3 online). Indeed, a comparison along all metro lines shows that velocity gradients calculated from Sentinel results tend to produce gradients values centred around zero, well within ± 0.1 $\times$ 10^-3^ year ^-1^, while those calculated from COSMO2 results spread more widely (Supplementary Fig. S4 online). Such discrepancy can be attributed to the fewer samples in space available from Sentinel results, which likely aliases subtle changes in the velocity signal, consequently smoothing the velocity gradient estimations.

We determine that COSMO2 results over Penón de los Baños and Peñón Viejo are reliable, in agreement with Sentinel results, and in contrast to TSX and COSMO1 results. Overall, COSMO1 results produce larger residuals when compared to any other dataset (Fig. 4b, d). Such larger residuals can be attributed to insufficient SAR scenes than the typically required for obtaining reliable PSI estimations (15–20 scenes)^23^. However, larger residuals over Penón de los Baños and Peñón Viejo produced by TSX, when compared to Sentinel and COSMO2 results (Fig. 4a, e), can be attributed to unwrapping errors. PSI algorithms perform unwrapping using wrapped phase values at PS locations after PS selection, which is based on phase stability estimations over time^24^. However, fast subsidence rates in the city are likely to produce unwrapping errors when fringe saturation is reached, as identified by previous works^16,22^.

### Unwrapping test over Peñón Viejo

In order to evaluate the impact of available PS samples on unwrapping over high-phase-gradient areas, we compare the wrapped phase values from both TSX and COSMO2 results at each dataset’s PS locations (Supplementary Fig. S5 online) and perform 1D unwrapping tests (Supplementary Fig. S6 online). We select Peñón Viejo as a test area and COSMO2 interferograms 2012/04/03-2012/05/13 (40 days) and 2012/04/03-2012/06/30 (88 days), and TSX interferograms corresponding to pairs 2012/02/06-2012/03/21 (44 days), 2012/02/06-2012/05/04 (88 days), and 2012/02/06-2012/10/27 (264 days). We observe higher PS density on the COSMSO2 interferograms around Peñón Viejo (Supplementary Fig. S5a and S5b online) as compared to TSX results (Supplementary Fig. S5c-e online). We attribute lower PS density around Peñón Viejo in the TSX results to the dataset’s longer time span (2 years versus 6 months of COMOS2 dataset, see Supplementary Table S3 online), at which phase likely loses stability over such non-urban areas.

The 1D unwrapping test using COSMO2 wrapped phase reveals a consistent signal buildup from 40 to 88 days (Supplementary Fig. S6a, b online). 1D unwrapping results of TSX 44- and 88-day interferograms should be very similar to the results of COSMO2 40- and 88-day interferograms. However, 1D unwrapping of TSX interferograms shows a phase range nearly as half as the results from COSMO2 (see Supplementary Fig. S6 online). Such unwrapped phase deficit persists in the case of the 264-day interferogram (see Supplementary Fig. S6e online ). We interpret that lower PS density in the TSX results, particularly around Peñón Viejo, produces phase aliasing leading to phase-unwrapping errors. In addition to having more phase samples due to its higher PS density around Peñón Viejo, the COSMO2 dataset has a shorter maximum temporal baseline (96 days, see Supplementary Table S3 online), which implies fewer wrapped phase cycles to be unwrapped. Consequently, phase unwrapping errors are less likely to occur in the COSMO2 dataset, producing reliable estimates over Peñón Viejo and Peñón de los Baños and other areas where high phase gradient is expected (see Fig. 3). TSX results, as well as COSMO1, could be improved by using higher resolution SAR data, other unwrapping and processing algorithms^25,26^ or data-fusion techniques^27^. Such improvements, nevertheless, go beyond the scope of this work.

## Discussion

In this section, we focus on the analysis of the InSAR results to conduct our geohazard assessment. To achieve this purpose, we first provide a summary of observations collected from the Results section. Additionally, we address further aspects concerning the reliability of the dataset and processing strategy selected to comprehensively characterize Mexico City’s subsidence, drawing on insights from published research. Subsequently, we interpret our results and evaluate the geohazard to the Metro system, integrating additional information and analyses from the literature. By elucidating potential mechanisms underlying the observed subsidence patterns, we contribute to a comprehensive understanding of the geohazard affecting the metro system.

### Validity of InSAR results

In the “Results” section, we presented four InSAR-derived vertical velocity estimates for Mexico City. We found that the datasets mostly agree among them. The main advantage of the X-band results is the higher sampling in space available from the datasets’ resolution, which is critical for our study. We also found that the main advantage of Sentinel results is that they have smaller uncertainties due to the abundance of SAR scenes used to calculate them over a longer period, which allowed us to use them as a benchmark to evaluate our X-band results. We observed that two of our X-band results (TSX and COSMO1) do not depict the stable behaviour of Peñón de los Baños and Peñón Viejo, which are two important areas for this study. Subsequently, we found that such behaviour can be attributed to unwrapping errors due to fringe saturation in the surroundings of such two isolated stable areas. After the comparison of all datasets, we identify that COSMO2 results provide the best estimate for the needs of our study. We list some of the arguments in favour of using COSMO2 results for our analysis: (1) COSMO2 dataset provides high spatial resolution (3m), (2) COSMO2 results fit well the longer-wavelength subsidence as compared to Sentinel results, (3) COSMO2 results capture the stable behaviour of Peñón de los Baños and Peñón Viejo, and (4) COSMO2 results provide complete spatial coverage of the Metro’s surface segments (Supplementary Fig. S2c online). Nevertheless, some questions may arise about whether or not the errors from comparing COSMO2 to Sentinel results are acceptable, or about possible nonlinearity, seasonality, or horizontal displacements affecting COSMO2 estimations. We clarify those questions in the following paragraphs.

A wealth of published works have evaluated the reliability of InSAR time series to characterize the subsidence process over Mexico City (see an extensive list in^17^). Day-to-day GPS versus Sentinel time series comparisons revealed an RMSE of 9 mm and a maximum difference in vertical velocities of 9 mm/yr in the 2017–2019 period^17^. Such error values are consistent with those obtained from ENVISAT versus GPS data in the 2004–2006 period, which are as high as 6.9 mm/year in the LOS direction^16^. Additionally, the analysis of the longest time series published to date, which uses roughly 24 years of InSAR data, 115 years of GPS data, and 14 years of levelling shows that the subsidence process has been highly linear since the 1950s^4^. Such highly-linear behaviour would produce lower errors for Sentinel estimates over longer time periods. Thus, we consider that the 2014–2020 Sentinel velocity estimates we use for validation are reliable, as we analyze a six-year period and would expect errors $\le$9 mm/year. Still, we obtain errors <24 mm/year from comparing COSMO2 with Sentinel vertical velocities. Nonetheless, previous studies using multiple InSAR-derived velocity maps over Mexico City reveal residuals <30 mm/year in across-algorithm comparisons^26^, and <50 mm/year in across-platform comparisons^4^. Since our comparison is both across algorithms and across platforms, and after we found Sentinel results to be a reliable solution, we determine that 24 mm/year represents a low error level, which implies that COSMO2 results constitute a reliable solution as well.

No significant seasonal displacements are present in Mexico City’s subsidence signal, as previously shown by GPS^4,16^ and the statistical analysis of a 6-year-long Sentinel-1 InSAR time series^4^. Hence, even though COSMO2 data were acquired over a 6-month period (Supplementary Tables S2 and S3 online), seasonal deformation is not expected to affect PSI velocity estimations. Even more, the 21 SAR scenes in the COSMO2 dataset are enough to estimate the atmospheric phase screen in the PSI processing, inasmuch as 15–20 scenes are typically required to obtain reliable measurements^23^. Furthermore, the 20 interferograms formed with the COSMO2 dataset (Supplementary Fig. S1c online) provide a reliable lower bound of deformation rate error, which is already as low as $\sim$0.3 mm/year^28^. Indeed, estimated velocity uncertainty levels of COSMO2 results are relatively small (0–5%)^19^. In addition, previous works have shown that the displacements due to subsidence in Mexico City are mostly in the vertical direction^4,16,17^. Therefore, we consider that the assumptions we make to obtain the vertical component of velocities in this work do not introduce any considerable error due to non-linearity, seasonality, or horizontal displacements of the subsidence process. Consequently, in the rest of the manuscript we use COSMO2 results to evaluate geohazard along the Metro lines.

### Geohazard evaluation of the Metro system

InSAR has been successfully applied to monitoring linear infrastructure worldwide (Supplementary Table S4 online). The main targets of such InSAR-bases studies have been elevated railways (e.g.^29,30^), surface railways (e.g.^27,31–36^), surface highways (e.g.^37,38^), and even shallow tunnels (e.g.^30,39^). Some studies have focused on analyzing not only displacement velocities but also seasonal variations over time (e.g.^27,40^). Only a few works have analyzed differential displacements using a post-processing technique, such as radius of curvature^31^, slope^31^ and entropy^39^, on $\sim$30-m resolution SAR datasets. In this work we have identified the need of using the highest-possible sampling in space. Accordingly, we here describe our geohazard evaluation based on a 3-m resolution SAR dataset.

**Figure 5:**
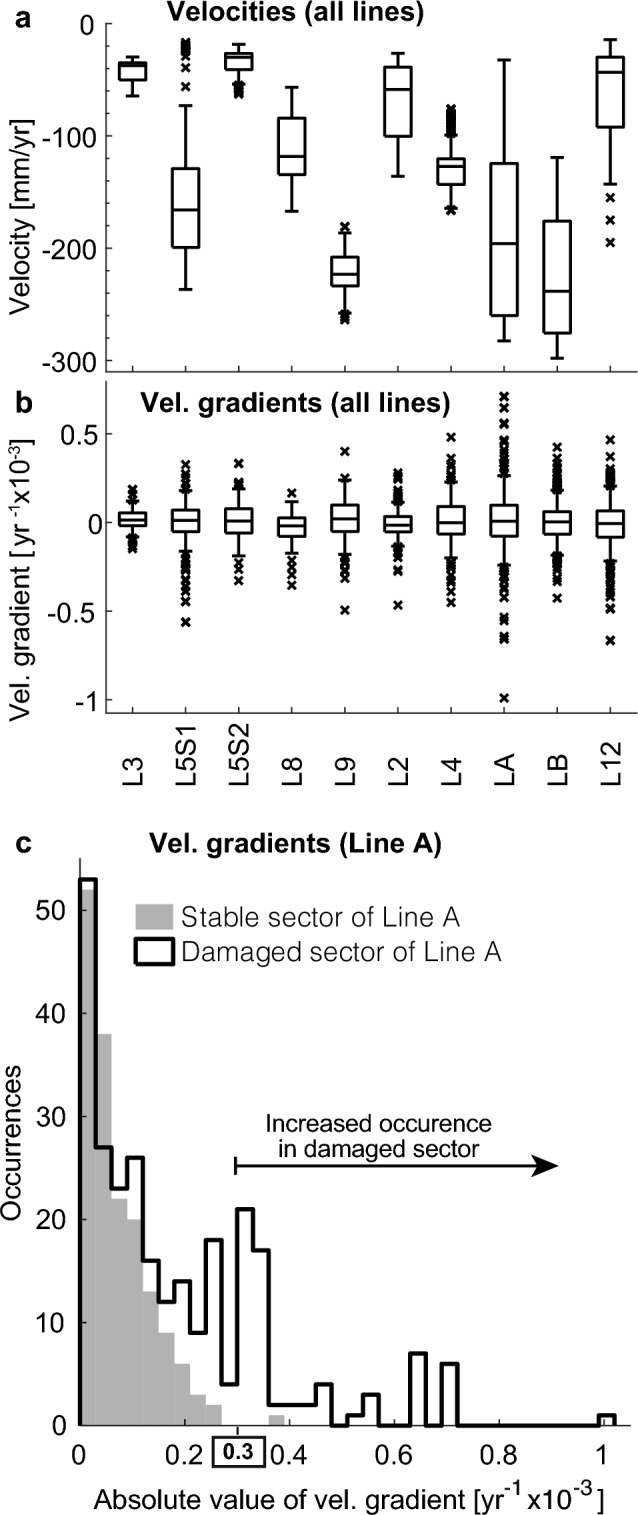
Distribution of velocities and velocity gradients as per COSMO2 results along the Metro system’s lines. (**a**) Velocities along all surface lines (see locations in Fig. 2c, velocities in Supplementary Fig. S3 online). (**b**) Calculated velocity gradients. Boxes are defined by the 1st and 3rd quartile of each dataset, the horizontal line within the boxes represents the median, whiskers delimit the 95% confidence interval, and x marks identify extreme values. (**c**) Overlain histograms showing velocity gradient’s absolute value distribution of two sectors of Line A (damaged sectors are indicated by a red patch in Fig. 2C and Supplementary Fig. S3 online). Figures created using Matlab R2015b (https://www.mathworks.com).

Subsidence velocities and velocity gradients vary greatly along Metro lines (Fig. 5a, b). The overall range of velocities along the system (0 to 310 mm/year) is significantly higher than those reported along linear infrastructure worldwide (Supplementary Table S4 online, Supplementary Fig. S7 online). However, velocities alone are not indicative of potential damage. For instance, L3 and L5S2 show subsidence velocities not greater than 80 mm/year (Fig. 5a). Still, velocity gradients of such two segments compare in range and magnitude to L8 and L9, which experience faster and/or a wider range of velocities (compare such two pairs in Fig. 5a, b). Therefore, significant subsidence velocities do not necessarily imply greater velocity gradients, and vice versa. Consequently, both subsidence velocities and velocity gradients can be used independently to evaluate different aspects of potential damage to the Metro infrastructure.

Consequences to the Metro’s infrastructure vary according to the type of exposed structure. For instance, LA, which is a surface segment running at street level, is likely to experience railway bending, whereas L12, which is an elevated segment, is likely to experience differential subsidence between columns. Our calculations might present limitations in comprehensively evaluating the damage to every type of structure, as we could calculate velocity gradients using a spacing greater or lesser than the 30 m we use (see “Materials and methods”). In fact, we identify railway bending occurring at distances shorter than 30 m (see Fig. 2d). Still, we consider that our calculations can present a fair oversight of the system at a broad scale. For instance, we find that velocity gradient values from segments of LA where damage has been reported present a distinct distribution as compared to stable sectors of the same line (Fig. 5c). In the following sections, therefore, we discuss the geohazard of the Metro system depending on the structure type and its exposure to subsidence and/or differential subsidence.

### Geohazard evaluation along street-level railways

**Figure 6:**
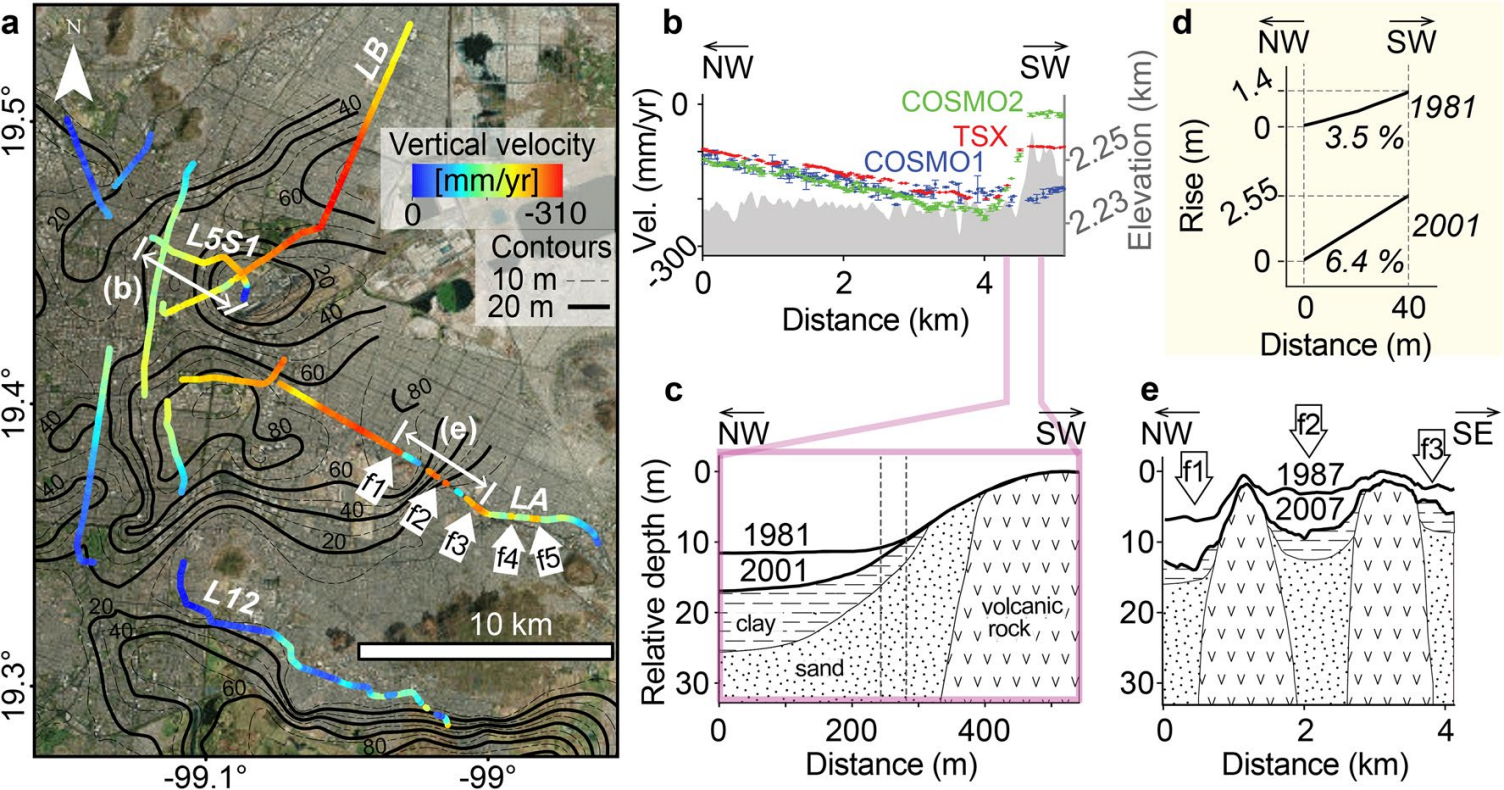
Subsidence along street-level and elevated lines of Mexico City Metro. (**a**) Vertical velocities along surface segments extracted from COSMO2 solution. Black contours represent the thickness of the uppermost clay-rich lacustrine lithological unit^8^. Thick white arrows indicate flood-prone areas from Fig. 2c. (**b**) Vertical velocities transect along street-level L5S1 from the 3 X-band SAR datasets. Grey shade shows topographic relief from SRTM (right axis). (**c**) Geologic section and railway level in 1981 and 2001 along a $\sim$500 m long section of L5S1^20^. Dashed thin vertical lines mark the location of (**d**). (**d**) Local measurement of the railway track slope along L5S1 between 1981 and 2001. (**e**) Inferred geologic section and railway level in 1987 and 2007 along a $\sim$4km segment of LA^21^, where f1–f3 arrows correspond to the flood-prone areas shown in (**a**). ArcMap 10.2 (https://www.esri.com/software/arcgis) was used to create the map, and Matlab R2015b (https://www.mathworks.com/) to create the figures. Satellite imagery credits: INEGI and Maxar Technologies.

Significant damage to the Metro system has occurred in the transition from stable to highly subsiding areas. Along L5S1 (Fig. 6a, b), COSMO2 results show that velocities vary from $-240$ mm/year (distance 4 km) to close to 0 mm/year in Peñón de los Baños (distance 4–5 km in Fig. 6b). Survey data from 1981 and 2001 reveal railway-elevation changes of up to 6 m, where the underlying geology corresponds to compressible sediments (Fig. 6c)^20^. Such elevation changes modified the railway’s slope to 6.4% (Fig. 6d), well beyond the maximum allowable slope of 3.5% established to ensure the safe operation of the trains’ braking systems^20^. During the 2015 train collision at this location (Oceanía Station in Fig. 2c), the terrain slope had reached 7%^41^ (Supplementary Text S1 online).

Increasing flooding hazards along LA recurrently led to trains’ shutdowns (e.g. 2013, 2016, 2018, 2021) (Supplementary Text S1). Flood report locations along LA (f1-f5 in Fig. 2c) match well with local minima of vertical velocities (i.e. faster velocities than their surroundings indicated by f1–f5 in Fig. 6a). Our findings are supported by field surveys from 1987 and 2007 (Fig. 6e)^21^, which revealed uneven stratigraphy-dependent topographic changes and most likely contributed to the occurrence of flooding along LA (f1–f3 in Fig. 6e).

**Figure 7:**
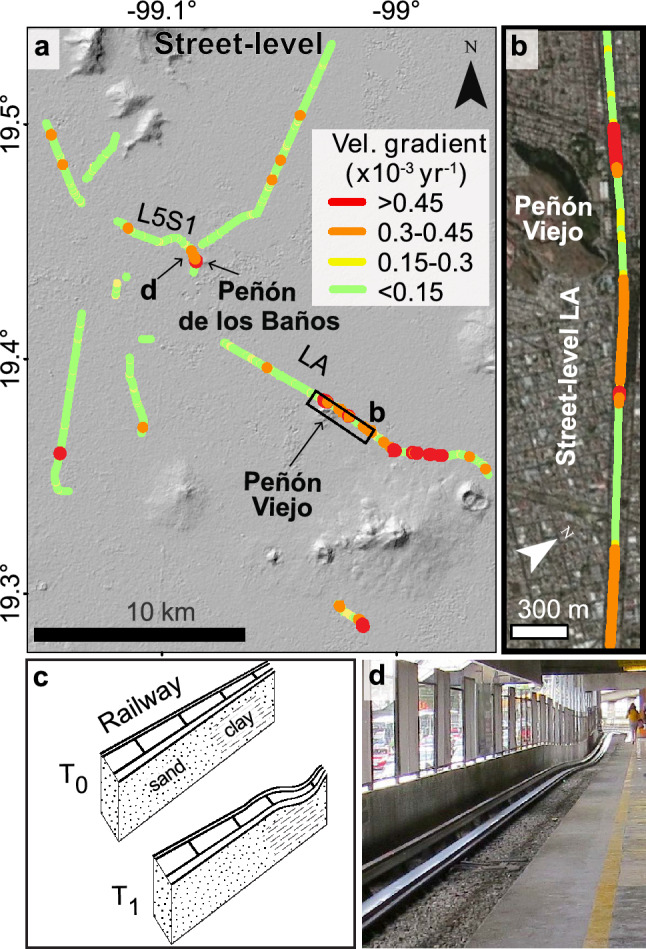
Velocity gradients along street-level railways of the Metro system. (**a**) Map of the calculated velocity gradients. The background is an SRTM hillshade map. The black rectangle indicates the extent of (**b**). (**b**) Detailed subsidence changes along LA in the vicinities of Peñón Viejo. (**c**) Schematic sections (not to scale) of railway track bending and its shallow geology. T_0_ and T_1_ are initial and final times, before and after deformation, respectively. (**d**) A photographic example of railway track deformation in Oceania station, close to Peñón de los Baños (location shown in (**a**)). ArcMap 10.2 (https://www.esri.com/software/arcgis) was used to create the maps. Shaded relief from SRTM data (https://earthexplorer.usgs.gov). Satellite imagery credits: INEGI and Maxar Technologies.

Based on the frequency distribution of velocity gradients along LA, we estimate that damaged sectors occur when velocity gradients >0.3 $\times 10^{-3}$ year^-1^. Particularly high gradients occur along LA, as in the northern flank of Peñón Viejo (>0.45 $\times 10^{-3}$ year^-1^) (Fig. 7a, b). Velocity gradient values become positive or negative depending on the direction in which they are calculated, but their absolute values represent the differential subsidence’s magnitude and serve as a pathfinder for damage correlation. The velocity gradients’ absolute values of both the stable and damaged sections of Line A (Fig. 5c) have modal classes close to zero; however, the damaged sector has an increased occurrence of values greater than $0.3 \times 10^{-3}$ year^-1^, clearly above the stable area’s gradients. We, thus, estimate that the velocity gradient threshold for problematic railway bending is $0.3 \times 10^{-3}$ year^-1^. Using this value as a damage-associated threshold for railway bending, we calculate that almost 6% of the street-level railway tracks of the entire Metro system are subjected to bending-induced damage.

### Geohazard evaluation along elevated viaducts

**Figure 8:**
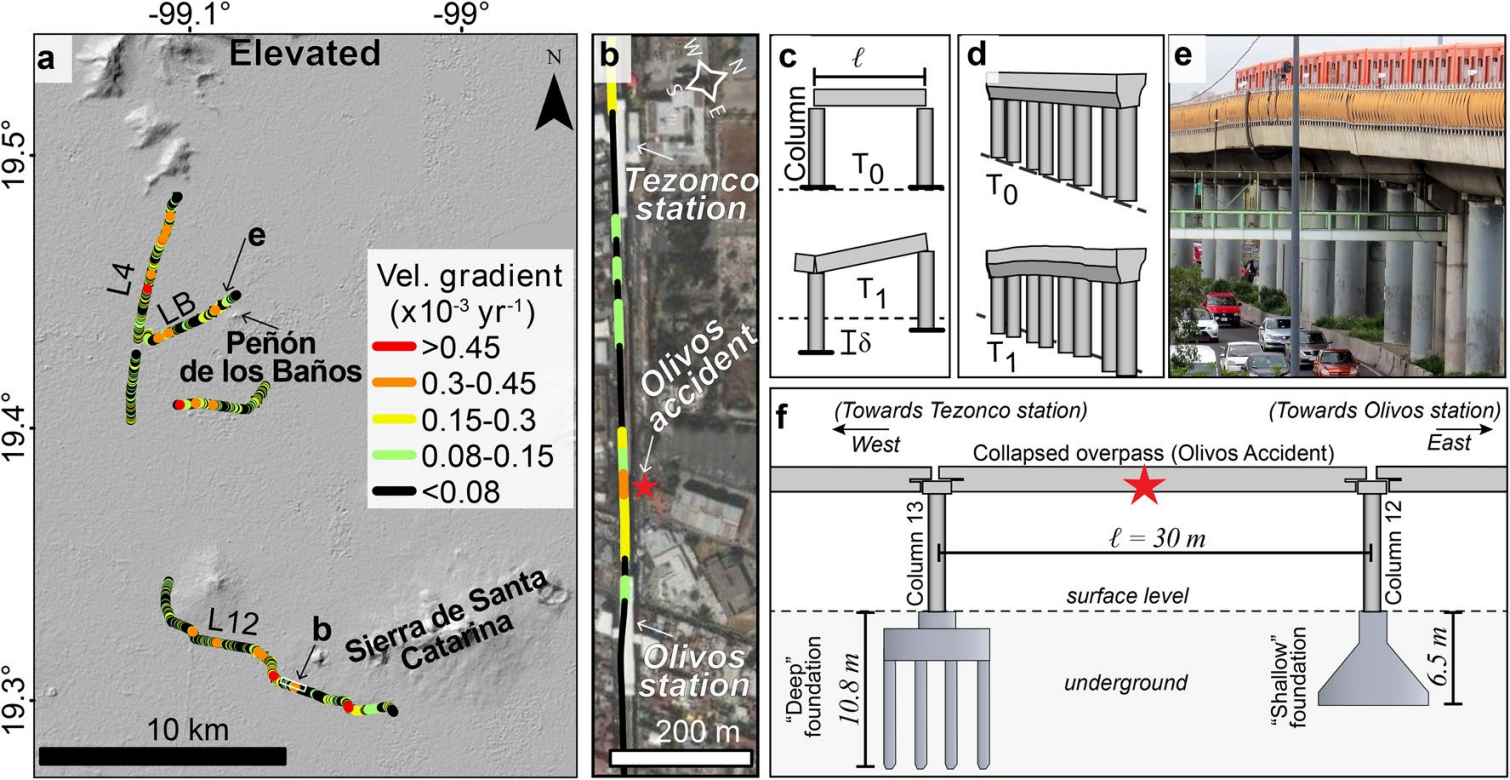
Velocity gradients along elevated viaducts of the Metro system. (**a**) Map of the calculated velocity gradients. The white frame indicates the extent of (**b**). (**b**) Zoom in to the location of the Olivos accident, as marked with a red star. Line segments are colour-coded according to the symbology in (**a**). (**c**) Schematic representation (not to scale) of differential settlement between columns, where $\delta$=differential settlement and $\ell$=distance between columns. T_0_ and T_1_ are before- and after-deformation times, respectively. (**d**) Schematic representation of the initial and final state of the deformation observed in (**e**). (e) Example of differential settlement between columns close to Peñón de los Baños (location shown in (**a**)). (**f**) Schematic representation of the structural design used in the overpass that collapsed during the Olivos accident, which occurred on May 3rd, 2021 (location indicated in (**b**)). Foundation design and dimensions were obtained according to^13,14^. ArcMap 10.2 (https://www.esri.com/software/arcgis) was used to create the maps. Shaded relief from SRTM data (https://earthexplorer.usgs.gov). Satellite imagery credits: INEGI and Maxar Technologies.

Here, we use velocity gradients to evaluate elevated segments of the metro lines (Fig. 8a, b). Differential subsidence between columns of elevated viaducts^42,43^ (Fig. 8c–e) compromises the stability of connecting slabs^44^. Differential subsidence between columns is typically evaluated with a parameter known as angular distortion^45^, also referred to as slope^46^, which is the ratio of the differential settlement between adjacent columns ($\delta$) and the distance between columns ($\ell$) (Fig. 8c). Distance between columns can be computed from the exact locations of every column in the metro system, which are not available to us. Still, we consider that the velocity gradients calculations in this work represent a smooth estimate of the angular distortion per year, assuming that the subsidence rates are constant over time, and that column spacing of 30 m reported along Line 12^13,47^ and measured from satellite imagery is consistent throughout the system.

In structural engineering analysis, service limit states (SLS) values indicate conditions at which structures become unsuitable for their intended use and require rehabilitation or repairs^44^. In the lake area of Mexico City, the official SLS value for angular distortion is 0.004^7^. Thus, we can calculate the time in which the SLS is reached-the SLS period-at each segment by dividing the official SLS value for angular distortion (0.004) by the corresponding velocity gradient value. According to the Metro’s design and development plans, SLS values should be not reached in periods shorter than 50 years^47^. However, we find that nearly 46% of the elevated segments experience velocity gradients >$0.08 \times 10^{-3}$ year^-1^ (Fig. 8a), which implies the need for serviceability before a 50-year period. Even though information on whether or not maintenance or rehabilitation has been performed on any given segment is not available to us, we are able to provide a proxy on locations most likely to have reached allowable angular distortion values. Several sectors along L4 with velocity gradient values >0.15 $\times 10 ^{-3}$ year^-1^, for instance, could have already reached the SLS since the line’s inauguration in 1982^48^.

### Differential displacements and the Olivos accident

On May 3rd, 2021, an overpass located approximately 200 m to the west of Olivos station along line 12 collapsed onto the road as a train was travelling over it^11,12^ (see location in Figs. 2c, 8b). Much controversy has been generated when trying to determine the causes of the collapse^49^. Root cause analyses on the incident^13,14^ indicated that the collapse occurred due to distortion and eventual fatigue of the overpass’ central transversal frame. Field evidence collected after the incident revealed important design and construction deficiencies in concrete and steel elements^13,14^. However, differential displacements between the columns supporting the collapsed overpass remain unquantified and unaccounted for in official evaluations, mainly due to the lack of permanent instrumentation measuring displacements and scarcity of pre-collapse levelling surveys in the area^13,14^. Here, we provide evidence suggesting that angular distortion might have been an additional contributing factor.

Forensic reports indicate that the collapsed overpass was supported by two columns, so-called columns 13 and 12 (Fig. 8f), located in a transition zone^13,14^. Even though the horizontal distance separating both columns is only 30 m, each column was built using a different foundation design^13,14^. Column 13 was built using a “deep” foundation composed of eight concrete piles reaching a depth of 10.8 m, whereas column 12 was supported by a “shallow” concrete footing reaching only 6.5 m deep (Fig. 8f)^13,14^. Post-collapse evaluations found no evidence of displacements between the columns’ foundations and the surrounding ground, and qualified both used foundation designs as adequate^13,14^.

Here, we observe a velocity gradient value of $0.37 \times 10 ^{-3}$ year^-1^ at the location of the May 3rd, 2021 Olivos accident (Fig. 8b). Such velocity gradients producing angular distortion since the line’s inauguration in 2012^48^ would imply a shortened service life of the collapsed structure. Indeed, the corresponding SLS period for angular distortion we calculate in such a segment would be 10.8 years-roughly a fifth of the intended 50-year service life. Admittedly, the averaging considered in our definition of velocity gradient (Eq. 1) produces a smoothened indicator of differential displacements, therefore leading to a conservative proxy for angular distortion along the 30 m segments we evaluate. Consequently, in-situ displacement measurements from field surveys at columns 12 and 13, as well as considerations for the exact distance separating columns, could provide even larger angular distortion values and shorter SLS periods.

Reaching an SLS, by itself, implies the need for repairs but does not necessarily imply that the structural stability of a well-designed well-constructed structure is compromised^44^. However, the collapsed structure was reportedly designed and constructed with plenty of deficiencies, including the use of poor-quality materials, the use of fewer reinforcement bolts, and incorrect welding techniques^13,14^. Under such conditions, angular distortion could have produced stress and strain on the structure’s elements, potentially affecting the materials’ properties and geometry for years. Repairs performed as early as January 2017, as well as inspections in December 2019, already revealed deformation in the overpass steel and concrete elements^13,14^. Clearly, an analysis incorporating the angular distortion values we quantify could improve the understanding of the structure’s conditions before and during the collapse.

**Figure 9:**
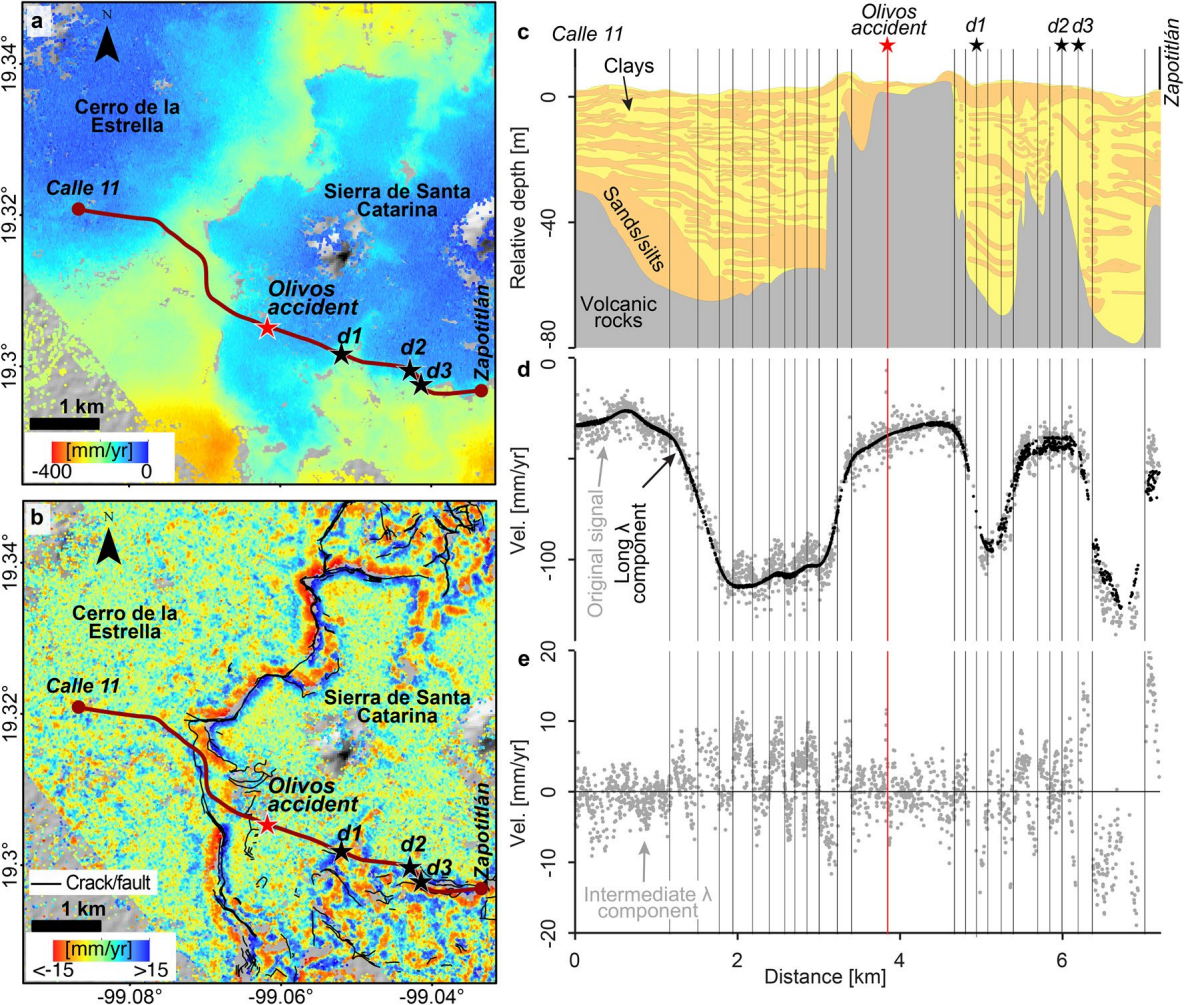
Subsidence and geotechnical context of the elevated segment of L12 across Sierra de Santa Catarina (modified from Fig. 4 in^19^). (**a**) Map of COSMO2 vertical velocities (extents indicated in Fig. 2c). The red star marks the location of the Olivos accident and the black stars mark the location of reported damage that occurred after the 2017 earthquakes in columns (d1^52^, d2^53^) and a connecting slab (d3^52^) of the elevated viaduct. (**b**) Intermediate wavelength component of the velocity map shown in (**a**), calculated according to^19^, overlaid by the traces of subsidence-related cracks/faults from^51^. Notice that the reported cracks/faults occur mainly along zero-crossings of the intermediate-wavelength component. The brown polyline in (**a**,**b**) indicates a transect from Calle 11 to Zapotitlán stations along L12 and is used for generating (**c–e**). (**c**) Geological section reinterpreted from^47,50^. (**d**) Velocity profile extracted from (**a**) and its corresponding long-wavelength component calculated according to^19^. (**e**) Intermediate-wavelength component velocities extracted from (**b**). In (**c–e**), vertical lines indicate the zero-crossings of the intermediate-wavelength component (**e**), where faulting occurs commonly, as reported in^19^.

Geotechnical evaluations following the collapse considered that variations in the underground’s geological composition and distribution did not influence the occurrence of the overpass collapse^13,14^. However, we present an analysis linking the occurrence of significant differential displacements and abrupt variation of the geological conditions over the transition area around Sierra de Santa Catarina, where the May 3rd, 2021 Olivos viaduct collapse took place. Displacements around Sierra de Santa Catarina have been quantified to be significant in the vertical direction^4,6,17,19^, and limited in space and magnitude in the horizontal direction^17^. In particular, Solano-Rojas et al.^19^ developed an approach to uncover hidden differential vertical displacement signals by obtaining three velocity components of long, intermediate, and short wavelengths using band-pass filtering on a high-resolution InSAR vertical velocity map. The intermediate-wavelength component approach has already revealed differential displacements in pedestrian overpasses of Pantitlán station and in columns of elevated lines 4 and B^19^ (locations in Fig. 2c). We apply such an approach to the COSMO2 vertical velocities over the Sierra de Santa Catarina area (Fig. 9a) (see “Materials and methods”) and present the map of the intermediate-wavelength component (Fig. 9b). We also present a reinterpreted geological section along L12^47,50^ (Fig. 9c) and its corresponding vertical velocities and long-wavelength component (Fig. 9d), as well as the intermediate-wavelength component (Fig. 9e).

The intermediate-wavelength component isolates differential displacements relevant to geotechnical and infrastructure monitoring^19^. Such component contains most of the misfit between the original velocity and the long-wavelength component (compare Fig. 9d, e) in what resembles a signal centred around zero with a varying amplitude over space, roughly within ±15 mm/year (Fig. 9b). Although stable areas corresponding to both Cerro de la Estrella and Sierra de Santa Catarina are surrounded by deforming areas, the intermediate-wavelength component shows signals only around Sierra de Santa Catarina (compare Fig. 9a, b). The geological section (Fig. 9c) shows a smooth geometry of the basal volcanic rocks westwards, towards Calle 11, compared to the complex geometry eastwards, towards Zapotitlán. The vertical velocities and their long-wavelength component mimic the general shape of the interface between volcanic and sedimentary rocks (Fig. 9d). The intermediate-wavelength component, however, shows a continuum behaviour towards Calle 11, but a more erratic behaviour and greater dispersion towards Zapotitlán (compare km 0–3.5 vs 3.5–7 in Fig. 9e). The intermediate-wavelength component, thus, seems to be determined by the compositional changes of the underground sedimentary deposits towards Calle 11 (i.e. Cerro de la Estrella), and by the basement’s geometry and the sediment layers’ thickness towards Zapotitlán (i.e. Sierra de Santa Catarina).

At the location where the Olivos collapse took place, the intermediate-wavelength component seems to indicate velocities around 0 mm/yr (see red vertical line in Fig. 9e). However, such velocities result from a change in the signal’s polarity (from positive to negative or vice versa) over a short distance, in what we term a zero-crossing. Such zero-crossing is consistent with the existence of considerable velocity gradient values at the collapse’s location. Zero-crossings also indicate other significant differential displacements^19^ and coincide with the location of cracks and faults mapped by the government^51^ (Fig. 9b). Zero-crossings along the Calle 11 and Zapotitlán transect, indicated by black vertical lines (Fig. 9c–e), have already been identified as potentially hazardous to infrastructure^19^. After the September 2017 earthquakes that affected Mexico City, important structural damage was reported in two columns of L12’s elevated bridges (indicated as d1^52^ and d2^53^ in Fig. 9a, c), as well as in one connecting slab (d3^52^ in Fig. 9a, c). All three reported features match the location of zero-crossings of the intermediate-wavelength component (black stars in Fig. 9b and black vertical lines in Fig. 9e, see labels in Fig. 9c). The detected hazardous condition in columns and connecting slab were derived from the COSMO2 dataset, which was acquired from 12/2011 to 06/2012, prior to the 2017 earthquake, and, therefore, free of co-seismic signals. We infer that the long-term effect of the differential displacements acting on the columns and slab at locations d1–d3 may have compromised their structural integrity, therefore exacerbating the effects of the strong motion induced by the September 2017 earthquakes. Admittedly, further verification and refinement of our city-wide assessment require ground-based observations, such as levelling surveys, geotechnical parameters obtained from laboratory tests, and geophysical surveys, as well as details on repairs and post-collapse improvements to structures, which are not available to us.

## Conclusions

Differential subsidence in Mexico City damages a significant length of the Metro railway tracks and is ultimately expressed as structural collapses, faults, cracks, track deformation, and slope changes, resulting in trains’ speed reduction and substandard performance, accidents, service interruptions, and loss of human life. Differential subsidence along the Metro system occurs because its tracks and related infrastructure spread over Mexico City’s largely heterogeneous geological setting, a portion of which undergoes fast differential land subsidence. Moreover, differential subsidence produces larger damage in the transition areas between stable volcanic rocks and rapidly subsiding surfaces. The powerful remote sensing approach we used to evaluate differential subsidence exploits space-based, high-resolution, synoptic-coverage geodetic measurements for a large-scale system evaluation and serves as a guidance aid for planning and conducting detailed, ground-based analysis. It will be beneficial to combine our methodology with ground-truth data and up-to-date high-resolution SAR data to plan maintenance and future line developments of the Metro infrastructure, given the Metro’s critical role for the mass transport in the city^10^. Still, a more detailed analysis should not consider as absolute the numerical values we present to evaluate railway bending potential or angular distortion but should incorporate ground truth data to determine thresholds more accurately in order to assert specific structural elements analysis and determine their tolerable deformation. Our analysis provides key information that can be incorporated in the root cause analysis of the Olivos accident, as well as in the evaluation of other vulnerable line segments. Furthermore, our analysis can provide insights into the future development of the Metro lines, such as LA’s 13-km expansion intended to reach Chalco^9^, considering that Chalco is subjected to very high subsidence rates (>350 mm/year) in proximity to the stable volcanic rock outcrops of Sierra de Santa Catarina (locations indicated in Fig. 2a). Finally, we recommend similar geohazard evaluations along other infrastructures features in Mexico City and other highly subsiding areas worldwide, particularly for Tehran’s metro system, where subsidence rates surpass 250 mm/year^36^ (Supplementary Fig. S7 online).

## Materials and methods

### Experimental design

In this study, we focus, first, on calculating displacements over Mexico City based on a wealth of satellite-acquired data and advanced InSAR techniques. Second, we focus on quantifying the displacements that affect the structures of the Metro system, for which we calculate average vertical velocities along the system. We then aim to quantify differential displacements along the Metro system, for which we calculate velocity gradients. We finally focus on obtaining a detailed scenario of the differential displacements around Sierra de Santa Catarina, where the May 3rd, 2021 Olivos viaduct collapse occurred, for which we use a band-pass filtering approach. In the following paragraphs, we provide details on the data we use and the calculations we perform.

### SAR data

Our study relies on 608 Synthetic Aperture Radar (SAR) scenes, which are divided into five datasets acquired by three satellite missions. Three of such datasets correspond to high-spatial-resolution ($\sim$3 m pixel size) X-band scenes acquired between 2011 and 2013 and over time windows of 4 and 6 months, and 2 years, whereas the two other datasets correspond to C-band scenes acquired from 2014 to 2020 with a lower spatial resolution ($\sim$15 m). We exploit both the high spatial resolution of the X-band datasets and the long-term velocity estimates of the C-band datasets.

One of the X-band datasets was acquired by the German TerraSAR-X satellite, while the other two were acquired by the Italian COSMO-SkyMed satellite constellation (Supplementary Table S2 online). The TerraSAR-X dataset, which we name TSX, consists of 34 scenes acquired along a single swath between May 2011 and June 2013 (Supplementary Table S3 online). The two COSMO-SkyMed datasets, which we name COSMO1 and COSMO2, were acquired along two nearby swaths with slightly different incident angles. COSMO1 consists of 15 scenes acquired from June 2011 to September 2011 and COSMO2 consists of 21 scenes acquired from December 2011 to June 2012 (Supplementary Table S3 online). In terms of covered area, COSMO1 and COSMO2 scenes cover 60% more area than TSX scenes (Fig. 2a). However, in terms of temporal coverage, the TSX dataset spans over $\sim$2 years, whereas COSMO1 and COSMO2 span over $\sim$4 and $\sim$6 months respectively (Supplementary Table S3 online).

The two C-band datasets amount to 538 SAR scenes acquired by the Sentinel-1A and B satellites from October 2014 to October 2020, from which 297 scenes correspond to track 143 in descending orbit and 241 scenes to track 78 in ascending orbit. Both C-band datasets have overlapping coverage over time and space, which allows us to observe the displacement velocities in the study area from two different radar geometries over the 6-year time windows.

### Overview of InSAR time series techniques

A variety of InSAR time series techniques have been developed in recent decades, and their capabilities have been systematically compared in several studies(e.g.^16,54–57^). The mentioned studies have conducted across-algorithm and across-dataset comparisons, and have determined that most time series InSAR techniques provide, generally, consistent results. However, such studies have also determined that no single technique has been found adequate for all situations. Here we provide a brief overview of the three main groups of existing time series, according to the characteristics of the scatters on the ground they exploit.

The first group of techniques exploits the scatterers with stable phase characteristics over time, which usually correspond to man-made features, and for which the trademarked Permanent Scatterers Interferometry technique was first developed^58^. An alternative to such a technique was later presented, which has the advantage of providing an openly available processing code, called the Stanford Method for Persistent Scatterers (StaMPS)^24^. The main advantage of PS techniques is that SAR scenes can be processed at full resolution, and the main setback is that the single-reference approach followed by this technique may lead to decorrelation due to temporal and geometrical baselines, which may limit the spatial sampling over non-urban areas^24,58,59^.

The second group of techniques exploits the so-called Distributed Scatterers (DS), which are identified as neighbouring pixels with similar behaviour. The pioneering technique exploiting DS avoided decorrelation by implementing a multi-reference approach and forming interferograms with short baselines^60^. The main advantage of using a short baselines approach is the decreased decorrelation, especially for datasets acquired over long time periods. An additional advantage is that using short temporal baselines avoids dealing with fringe saturation due to signals varying greatly over short distances. The main disadvantage of an SBAS approach is the reduced spatial resolution of the dataset due to its required multi-looking and the potential bias in the velocity estimations^61^. The Miami INsar Time-series software in Python (MintPy)^62^ is an openly available code for processing such an approach.

The third group of techniques considers a joint use of PS and DS based on a statistical treatment of their characteristics. Squeesar is the pioneering but proprietary algorithm for this group of techniques^63^. The openly-available alternative code is the very recently released MIAmi Phase Linking software in Python (MiaplPy)^64^. While this group of techniques has been demonstrated to provide better accuracy, their implementation implies a considerable increase in computational resources, which is an important aspect to consider in the big SAR data era (e.g.^61^). This group of techniques provides a very high density of spatial samples, which represents a critical advantage over non-urban areas. However, the application of these techniques falls beyond the scope of our study on the urban area of Mexico City.

### InSAR time series processing

We process the three X-band datasets using a PSI approach, as it focuses on stable-phase targets smaller than the SAR resolution cell and allows the processing of SAR data at full resolution, therefore maximizing the spatial sampling of the resulting velocity maps. The PSI algorithm typically calculates interferograms using a single-master approach, which is suitable for the short time span of the three X-band datasets but might lead to important fringe saturation in the 6-year acquisition period of the C-band datasets^26^. The revisit time of the C-band dataset, however, is of up to 6 days, which allows the formation of interferograms with very short temporal baselines to prevent fringe saturation. Consequently, we choose to process the C-band datasets with a multi-master interferogram network using an SBAS approach^60^.

We used a two-step procedure for producing the X-band InSAR velocity maps. In the first step, we generated interferograms using the Delft object-oriented radar interferometric software (Doris)^65^. The interferograms were produced using a single-master approach, and the topographic phase was corrected using a 30 m SRTM DEM^66^. We selected the master scene for each dataset aiming to minimize perpendicular and temporal baselines (Supplementary Fig. S1A–C online, Supplementary Table S3 online). In the second step, we calculated the Line-of-Sight (LOS) displacement velocities based on the PSI approach^58^ using StaMPS^24^, which is one of the most used algorithms for InSAR time series analysis^26^. The mean PS densities obtained were 4827, 3078, and 3879 PS/km^2^ for TSX, COSMO1, and COSMO2 datasets, respectively (Supplementary Table S3 online).

Previous research using InSAR and GPS indicates that displacements in the city are predominantly vertical^16,67^; thus, we assume that the LOS velocities we obtain mainly result from vertical displacements. Therefore, for each velocity map, we projected the LOS velocities using the following expression V_vert_=V_LOS_/cos(i), where V_vert_ is the vertical velocity, V_LOS_ is the LOS velocity, and i is the mean incidence angle of each dataset (Supplementary Table S3 online). The calculated vertical velocities are presented in Fig. 3a–c.

We processed the C-band datasets in two steps. First, we use the InSAR Scientific Computing Environment (ISCE)^68,69^) using a three-neighbor design to obtain a fully-connected network of interferograms (Supplementary Fig. S1D, S1E online). We multilooked the SAR images to get a pixel size of $\sim$30 m, and perform a correction for topographic phase using a 30 m SRTM DEM^66^. Second, we use the SBAS approach^60^ implemented in the Miami INsar Time-series software in PYthon (MintPy)^62^ to calculate the LOS displacement from both datasets. The calculated velocity maps are presented in Supplementary Fig. S3D, S3E online. We determine the displacement in the vertical direction^70^ by combining both ascending and descending LOS velocity maps, as implemented in MintPy, given that two different observation geometries are available. The calculated vertical velocities are presented in Fig. 3f.

### Velocity gradient calculation

Based on the COSMO2 velocity map (Fig. 3c), we calculated directional velocity gradients to evaluate differential subsidence along the surface Metro lines. We excluded underground segments because no information on the depth and trace of such segments is available. The concept of directional gradients considers changes along a given direction over a surface; thus, we use this approach to identify areas subjected to differential subsidence along the Metro lines running at the surface level, potentially leading to damage and malfunctioning. Additionally, we use the directional velocity gradient calculations to evaluate damage-prone areas due to differential subsidence along the surface railways considering the constructive design of both street-level and elevated segments.

Velocity gradient calculations directly from PS could misrepresent differential displacements at specific locations due to variable PS density (typically spaced every 3–15 m) and measurement noise. Therefore, we discretize the surface lines, assign an average subsidence velocity value per discretized segment, and then calculate their gradients. Our procedure includes the following steps:

(i) Metro lines discretization. We identify the segments of the Metro running on the surface (elevated and street-level segments) by using online-available digitized traces of the Metro system’s network^71^ and high-resolution DigitalGlobe imagery. Technical reports^13^ and satellite imagery show that columns supporting elevated viaducts are spaced every 30 m. Therefore, we select such distance to discretize all metro lines.
(ii) Average subsidence velocities calculation along the Metro lines. We calculate and assign an average velocity for each discretized segment considering the PS within a 30 m by 10 m rectangle because 10 m is the average railroad width in the system as measured from orthorectified DigitalGlobe images. The average subsidence velocity is obtained from every PS within the discretized segment’s rectangle. In rectangles where data density is insufficient, we apply linear interpolation using the neighbouring segments’ velocities.
(iii) Velocity gradients calculation. We calculate the gradients for every segment using the following relation:
  1 $$\begin{aligned} S_{i} = \frac{1}{2} \left( \frac{v_{i-1}-v_{i}}{|x_{i-1}-x_{i}|} + \frac{v_{i}-v_{i+1}}{|x_{i}-x_{i+1}|}\right) \end{aligned}$$

Where $S_i$ is the average velocity gradient at the *i*-th segment, $v_{i-1}$, $v_i$ and $v_{i+1}$ are the velocities at the *i*-1, *i*, and the *i*+1 segments, and $x_{i-1}$, $x_i$, $x_{i+1}$ are the distances from one of the endpoints of the Metro line to the centre of the *i*-1, *i*, and the *i*+1 segments.

### Band-pass filtering

We follow the band-pass filtering approach implemented by^19^ on the COSMO2 vertical velocity map over Sierra de Santa Catarina (Fig. 9a, extents indicated in Fig. 2c) to obtain components of long (>478 m), intermediate (42–478 m), and short (<42 m) wavelengths. A complete description of the implementation of the method can be found in^19^ and its Supplementary information. The filtering approach can be summarized as follows: (1) 2D Fast Fourier Transform (FFT) calculation of an InSAR vertical velocity map, (2) power spectrum analysis and low-spatial-frequency threshold ($D_1$) determination, (3) signal analysis in the spatial domain and high-spatial-frequency threshold ($D_2$) determination, (4) filter design and filtering in the spatial-frequency domain, and (5) retrieval of the signal components in the spatial domain by using inverse Fourier transform (IFFT).

We use the COSMO2 vertical velocity map as input. We obtain and centre its 2D Fourier transform *F*(*u*, *v*), and calculate its distance function *D*(*u*, *v*), where *u*and *v* are the spatial frequencies in the longitude and latitude directions^72^. Solano-Rojas et al. already determined $D_1$ and $D_2$ thresholds for the study area to be $D_1= 478$ m $= 2.095 \times 10$ ^-3^ cycles/m and $D_2$= 42 m= $23.809 \times 10^{-3}$ cycles/m^19^. Therefore, we use such thresholds and the distance function *D*(*u*, *v*) to design second-order band-pass Butterworth filters $H_1$, $H_2$, $H_3$^73^:

2 $$\begin{aligned} H_{1}(u,v)= & {} 1/(1 + [D(u,v)/D_{1}]^4) \end{aligned}$$

3 $$\begin{aligned} H_{2}(u,v)= & {} 1 - (1/(1 + [D(u,v)/D_{2}]^4)) \end{aligned}$$

4 $$\begin{aligned} H_{3}(u,v)= & {} (1-H_1) * (1-H_2) \end{aligned}$$

We then obtain the frequencies’ bands $G_1$, $G_2$ and $G_3$ using $H_1$, $H_2$, $H_3$ and *F*(*u*, *v*) according to the Eq. 5^74^:

5 $$\begin{aligned} G(u,v) = H(u,v)F(u,v) \end{aligned}$$

Finally, we calculate the 2D IFFT of $G_1$, $G_2$ and $G_3$ to obtain the components of long, intermediate, and short wavelengths. We present a map of the intermediate-wavelength component in Fig. 9b.

### Supplementary Information

Supplementary Information 1.

Supplementary Information 2.

## Data Availability

SAR data were provided by the German Space Agency (DLR), the Italian Space Agency (ASI), and the European Space Agency (ESA). Code for the velocity gradient analysis is available from the authors upon direct request to Darío Solano-Rojas.

## References

[1] Sistema de Transporte Colectivo. *Cifras de Operación del Metro*. http://www.metro.cdmx.gob.mx/operacion/cifras-de-operacion (2017).

[2] Crôtte A, Noland RB, Graham DJ (2009). Is the Mexico City metro an inferior good?. Transport Policy.

[3] Gambolati G, Teatini P (2015). Geomechanics of subsurface water withdrawal and injection. Water Resour. Res..

[4] Chaussard E, Havazli E, Fattahi H, Cabral-Cano E, Solano-Rojas D (2021). Over a century of sinking in Mexico City: No hope for significant elevation and storage capacity recovery. J. Geophys. Res. Solid Earth.

[5] Santoyo-Villa, E., Ovando-Shelley, E., Mooser, F. & Leon-Plata, E. *Síntesis Geotécnica de la Cuenca del Valle de Mexico*. Vol. 1. 1st edn arXiv:1011.1669v3 (Publicaciones TGC, 2005).

[6] Cabral-Cano E (2008). Space geodetic imaging of rapid ground subsidence in Mexico City. Bull. Geol. Soc. Am..

[7] Gobierno del Distrito Federal. *Normas técnicas Complementarias para Diseño y Construcción de Cimentaciones* (2004).

[8] Solano-Rojas D (2015). La relación de subsidencia del terreno InSAR-GPS y el abatimiento del nivel estatico en pozos de la zona Metropolitana de la Ciudad de Mexico. Bol. Soc. Geol. Mexicana.

[9] Sistema de Transporte Colectivo. Fideicomiso maestro del Metro: Once compromisos asumidos por el Sistema de Transporte Colectivo. In *Technical Report* (2014).

[10] Sistema de Transporte Colectivo (2018). Technical Report.

[11] Jefatura de Gobierno de la Ciudad de México. Atiende Gobierno de la Ciudad de México y Gobierno de México incidente en L-12 del Metro. https://www.jefaturadegobierno.cdmx.gob.mx/comunicacion/nota/informa-gobierno-capitalino-que-se-realizara-dictamen-de-todos-los-tramos-elevados-de-la-linea-12-del-metro (2021).

[12] Jefatura de Gobierno de la Ciudad de México. Informa Gobierno capitalino que se realizará dictamen de todos los tramos elevados de la Línea 12 del Metro. https://www.jefaturadegobierno.cdmx.gob.mx/comunicacion/nota/atiende-gobierno-de-la-ciudad-de-mexico-y-gobierno-de-mexico-incidente-en-l-12-del-metro (2021).

[13] DNV. Dictamen técnico del incidente ocurrido en la línea 12 en el tramo elevado entre las estaciones Olivos y Tezonco, entre las columnas 12 y 13, y análisis de causa-raíz. Dictamen final fase II. In *Technical Report, DNV Energy Systems Mexico S. de R.L. de C.V., Mexico City*. https://www.proteccioncivil.cdmx.gob.mx/storage/app/media/Dictamen/DICTAMEN_FINAL_07_09_2021_12_50.pdf (2021)

[14] DNV. Dictamen técnico del incidente ocurrido en la línea 12 en el tramo elevado entre las estaciones Olivos y Tezonco, entre las columnas 12 y 13, y análisis de causa-raíz. Dictamen preliminar fase In *Technical Report, DNV Energy Systems Mexico S. de R.L. de C.V., Mexico City* https://proteccioncivil.cdmx.gob.mx/storage/app/uploads/public/60c/a54/3b4/60ca543b4ac47042812476.pdf (2021).

[15] CDMX, G. *Anexo Técnico Términos de Referencia del Proyecto de Prestación de Servicios a Largo Plazo denominado “Modernización Integral de la Línea 1 del Sistema de Transporte Colectivo*”. https://www.proyectosmexico.gob.mx/wp-content/uploads/2020/03/Anexo-1-Anexo-T%C3%A9cnico.pdf (2020).

[16] Osmanoğlu B, Dixon TH, Wdowinski S, Cabral-Cano E, Jiang Y (2011). Mexico City subsidence observed with persistent scatterer InSAR. Int. J. Appl. Earth Obs. Geoinf..

[17] Cigna F, Tapete D (2021). Present-day land subsidence rates, surface faulting hazard and risk in Mexico City with 2014–2020 Sentinel-1 IW InSAR. Remote Sens. Environ..

[18] Fernández-Torres, E., Cabral-Cano, E., Solano-Rojas, D., Havazli, E. & Salazar-Tlaczani, L. Land subsidence risk maps and InSAR based angular distortion structural vulnerability assessment: an example in Mexico City. In *Proceedings IAHS*. Vol. 382. 583–587. 10.5194/piahs-382-583-2020 (2020).

[19] Solano-Rojas D, Wdowinski S, Cabral-Cano E, Osmanoğlu B (2020). Detecting differential ground displacements of civil structures in fast-subsiding metropolises with interferometric SAR and band-pass filtering. Sci. Rep..

[20] Escamilla Estrada, A. & Palacios Sánchez, E. *Hundimiento regional del Valle de México y el STC (Estudio de caso, tramo terminal aérea-oceanía de la línea 5 del metro)*. Thesis, Universidad Nacional Autónoma de México http://132.248.52.100:8080/xmlui/handle/132.248.52.100/15041 (2009).

[21] Mora Ramírez, P. J. *Trabajo topográfico y geodésico en el tramo Guelatao-Los Reyes de la línea Pantitlán-La Paz del Sistema del Transporte Colectivo (STC) de la Ciudad de México*. Thesis, Universidad Nacional Autónoma de México http://132.248.52.100:8080/xmlui/handle/132.248.52.100/9737%09 (2016).

[22] Yan Y (2012). Mexico City subsidence measured by InSAR time series: Joint analysis using PS and SBAS approaches. IEEE J. Sel. Top. Appl. Earth Obs. Remote Sens..

[23] Marinkovic PS, Van Leijen F, Ketelaar G, Hanssen RF (2005). Recursive data processing and data volume minimization for PS-InSAR. Int. Geosci. Remote Sens. Sympos. (IGARSS).

[24] Hooper A, Segall P, Zebker H (2007). Persistent scatterer interferometric synthetic aperture radar for crustal deformation analysis, with application to Volcan Alcedo, Galapagos. J. Geophys. Res. Solid Earth.

[25] Crosetto M, Monserrat O, Cuevas-González M, Devanthéry N, Crippa B (2015). Persistent scatterer interferometry: A review. ISPRS J. Photogramm. Remote Sens..

[26] Osmanoğlu, B., Sunar, F., Wdowinski, S. & Cabral-Cano, E. Time series analysis of InSAR data: Methods and trends. *ISPRS J. Photogramm. Remote Sens.* (2016).

[27] Wang Y, Zhu XX, Zeisl B, Pollefeys M (2016). Fusing meter-resolution 4-D InSAR point clouds and optical images for semantic urban infrastructure monitoring. IEEE Trans. Geosci. Remote Sens..

[28] Adam N, Parizzi A, Eineder M, Crosetto M (2009). Practical persistent scatterer processing validation in the course of the Terrafirma project. J. Appl. Geophys..

[29] Hung WC (2009). Monitoring severe aquifer-system compaction and land subsidence in Taiwan using multiple sensors: Yunlin, the southern Choushui river Alluvial fan. Environ. Earth Sci..

[30] Perissin, D., Wang, Z. & Lin, H. Shanghai subway tunnels and highways monitoring through Cosmo-SkyMed persistent scatterers. *ISPRS J. Photogramm. Remote Sens.* **73**, 58–67 http://www.sciencedirect.com/science/article/pii/S0924271612001256. 10.1016/j.isprsjprs.2012.07.002 (2012).

[31] GuangYao D (2016). Monitoring and analysis of land subsidence along Beijing–Tianjin inter-city railway. J. Indian Soc. Remote Sens..

[32] Zhang, H., Tao, L., Wang, C. & Tang, Y. X. Ground deformation detection along Beijing–Tianjin intercity railway using advanced network multi-baseline DInSAR. In *Procedings of the 2010 International Conference on Wavelet Analysis and Pattern Recognition, ICWAPR 2010*, July, pp. 222–226. 10.1109/ICWAPR.2010.5576330 (2010).

[33] Ge, D. *et al.* Using permanent scatterer Insar to monitor land subsidence along high speed railway-the first experiment in China. In *Fringe 2009 Workshop, Frascati, Italy*. Vol. 2009 (2009).

[34] Galve JP, Castañeda C, Gutiérrez F (2015). Railway deformation detected by DInSAR over active sinkholes in the Ebro Valley evaporite karst, Spain. Nat. Hazards Earth Syst. Sci..

[35] Chen F, Lin H, Li Z, Chen Q, Zhou J (2012). Interaction between permafrost and infrastructure along the Qinghai–Tibet railway detected via jointly analysis of C- and L-band small baseline SAR interferometry. Remote Sens. Environ..

[36] Haghshenas, H.M. & Motagh, M. Ground surface response to continuous compaction of aquifer system in Tehran, Iran: Results from a long-term multi-sensor InSAR analysis. *Remote Sens. Environ.* **221**, 534–550. https://linkinghub.elsevier.com/retrieve/pii/S003442571830504210.1016/j.rse.2018.11.003 (2019).

[37] Wu, H., Zhang, Y., Zhang, J. & Chen, X. Mapping deformation of man-made linear features using DInSAR technique. In *ISPRS TC VII Symposium—100 Years ISPRS*. Vol. XXXVIII. 293–297 (2010).

[38] Shi, X. *et al.* Expressway deformation mapping using high-resolution TerraSAR-X images. *Remote Sens. Lett.* **5**, 194–203 10.1080/2150704X.2014.891774 (2014).

[39] Chen, W.-F. *et al.* Spatiotemporal evolution of land subsidence around a subway using InSAR time-series and the entropy method. *GISci. Remote Sens.*10.1080/15481603.2016.1257297 (2016).

[40] Karimzadeh S, Matsuoka M, Ogushi F (2018). Spatiotemporal deformation patterns of the Lake Urmia Causeway as characterized by multisensor InSAR analysis. Sci. Rep..

[41] Excelsior. *Deja 12 Lesionados Choque de Trenes en Estación Oceanía* http://www.excelsior.com.mx/comunidad/2015/05/04/1022331#imagen-1 (2015).

[42] Llanos, R. *Cinco líneas del Metro, Afectadas por Hundimientos Diferenciales: Bojórquez* (2010). http://www.jornada.unam.mx/2010/06/17/capital/033n1cap

[43] Hernández, A. *Crecen Grietas en Paredes y Pisos del Metro Pantitlán* http://www.eluniversal.com.mx/articulo/metropoli/df/2015/07/11/crecen-grietas-en-paredes-y-pisos-del-metro-pantitlan (2015).

[44] AASHTO. *Standard Specifications for Highway Bridges*. 6th edn (AASHTO, 2012).

[45] Skempton, A. W. & MacDonald, D. H. The allowable settlements of buildings. In *Proceedings of the Institution of Civil Engineers*. Vol. 5. 727–768 (Thomas Telford-ICE Virtual Library, 1956).

[46] Polshin, D. E. & Tokar, R. A. Maximum allowable non-uniform settlement of structures. In *Proceedings of the 4th International Conference on Soil Mechanics and Foundation Engineering*. Vol. 1. 402–405 (Butterworth’s London, 1957).

[47] Rodriguez, G. & Soria, C. Comportement du viaduc élevé de la ligne 12 du métro de la Ville de Mexico. In *Proceedings of the 18th International Conference on Soil Mechanics and Geotechnical Engineering*. Vol. 1. 1345–1348 (2013).

[48] Sistema de Transporte Colectivo. *Inauguraciones y Ampliaciones en Orden Cronológico* https://www.metro.cdmx.gob.mx/cronologia-del-metro (2017).

[49] Informe de las Inconsistencias y Contradicciones Técnicas y Científicas del Reporte del Análisis de Resultados de Causa-raíz Elaborado por DNV Fase III NO Aceptado por la Secretarííía de Gestión Integral de Riesgos y Protección Civil https://transparencialinea12.cdmx.gob.mx/storage/app/media/Observaciones%20SGIRPC/dnv-presentacion-final-2.pdf (2022).

[50] Limaymanta, F. M., García, S. & Pliego, L. F. Delimitación neuronal de zonas geológicas usando ruido sísmico : Suelos de Transición en la línea 12 del Metro. In *XXVI Reunión Nacional de Mecánica de Suelos e Ingeniería Geotécnica*. 1–8 (Sociedad Mexicana de Ingeniería Geotécnica, A.C., 2012).

[51] Centro Nacional de Prevención de Desastres (CENAPRED). *Fracturas en la Ciudad de Mexico* http://www.atlasnacionalderiesgos.gob.mx/archivo/visor-capas.html (2017).

[52] Sistema de Transporte Colectivo. *Afectaciones por Sismo en Línea 12*. https://www.metro.cdmx.gob.mx/acerca-del-metro/mas-informacion/informacion-linea-12/afectaciones-en-la-red-del-metro (2017).

[53] Hernández, E. *Vecinos temen colapso de Línea 12 del Metro por Sismo*. https://www.eluniversal.com.mx/metropoli/cdmx/vecinos-temen-colapso-de-linea-12-del-metro-por-sismo (2017).

[54] Even M, Schulz K (2018). InSAR deformation analysis with distributed scatterers: A review complemented by new advances. Remote Sens..

[55] Minh DHT, Hanssen R, Rocca F (2020). Radar interferometry: 20 years of development in time series techniques and future perspectives. Remote Sens..

[56] Karamvasis K, Karathanassi V (2020). Performance analysis of open source time series InSAR methods for deformation monitoring over a broader mining region. Remote Sens..

[57] Sadeghi Z (2021). Benchmarking and inter-comparison of Sentinel-1 InSAR velocities and time series. Remote Sens. Environ..

[58] Ferretti A, Prati C, Rocca F (2001). Permanent scatters in SAR interferometry. IEEE Trans. Geosci. Remote Sens..

[59] Hooper A, Zebker H, Segall P, Kampes B (2004). A new method for measuring deformation on volcanoes and other natural terrains using InSAR persistent scatterers. Geophys. Res. Lett..

[60] Berardino P, Fornaro G, Lanari R, Sansosti E (2002). A new algorithm for surface deformation monitoring based on small baseline differential SAR interferograms. IEEE Trans. Geosci. Remote Sens..

[61] Ansari H, De Zan F, Parizzi A (2021). Study of systematic bias in measuring surface deformation with SAR interferometry. IEEE Trans. Geosci. Remote Sens..

[62] Yunjun Z, Fattahi H, Amelung F (2019). Small baseline InSAR time series analysis: Unwrapping error correction and noise reduction. Comput. Geosci..

[63] Ferretti A (2011). A new algorithm for processing interferometric data-stacks: Squeesar. IEEE Trans. Geosci. Remote Sens..

[64] Mirzaee S, Amelung F, Fattahi H (2023). Non-linear phase linking using joined distributed and persistent scatterers. Comput. Geosci..

[65] Kampes, B. M., Hanssen, R. F. & Perski, Z. Radar interferometry with public domain tools. In *European Space Agency (Special Publication) ESA SP*. 59–68 (2004).

[66] Farr TG (2007). The shuttle radar topography mission. Rev. Geophys..

[67] Chaussard E, Wdowinski S, Cabral-Cano E, Amelung F (2014). Land subsidence in central Mexico detected by ALOS InSAR time-series. Remote Sens. Environ..

[68] Rosen, P. A., Gurrola, E., Sacco, G. F. & Zebker, H. The InSAR scientific computing environment. In *9th European Conference on Synthetic Aperture Radar, 2012. EUSAR. * 730–733 (2012).

[69] Fattahi H, Agram P, Simons M (2017). A network-based enhanced spectral diversity approach for TOPS time-series analysis. IEEE Trans. Geosci. Remote Sens..

[70] Wright TJ, Parsons BE, Lu Z (2004). Toward mapping surface deformation in three dimensions using InSAR. Geophys. Res. Lett..

[71] Archambault, R. *The Complete System on Google earth (KML)*. http://www.mexicometro.org/Mexico-Metro.kmz (2016).

[72] González, R. C., Woods, R. E. & Eddins, S. L. *Digital Image Processing Using MATLAB *. 109–140 (Pearson/Prentice Hall, 2004).

[73] Butterworth, S. *On the Theory of Filter Amplifiers* (1930).

[74] Najim M (2006). Digital Filters Design for Signal and Image Processing.

[75] Gobierno de la Ciudad de México. Reportes de encharcamientos 2016-2018 del atlas de riesgo de la CDMX. In *Technical Report, Gobierno de la Ciudad de México, Mexico City*. http://atlas.cdmx.gob.mx/analisisn2/ (2018).

[76] Redacción. *Línea A Reanuda Servicio Tras Inundación en Vías por Fuertes Lluvias* (2021).

