# Supplementary material for: Geohazard assessment of Mexico City’s Metro system derived from SAR interferometry observations
Source: Sci Rep. 2024 Mar 12;14:6035. doi: 10.1038/s41598-024-53525-y (PMC10933379; doi:10.1038/s41598-024-53525-y)
Supplement: Supplementary file 2 — Supplementary Information 2. [file 41598_2024_53525_MOESM2_ESM.pdf]

Supplementary Materials for

Geohazard assessment of Mexico City's Metro system derived from SAR interferometry observations

Darío Solano-Rojas[1,2,3*], **Shimon Wdowinski**[3], **Enrique Cabral-Cano**[4], **and Batuhan Osmanoğlu.**[5]

[1]División de Ingeniería en Ciencias de la Tierra, Facultad de Ingeniería, Universidad Nacional Autónoma de México, México CDMX, 04360, México.
[2]Department of Earth and Environment, Institute of Environment, Florida International University, Miami, FL 33199, USA.
[3]School of Marine and Atmospheric Science, University of Miami, 4600 Rickenbacker Causeway, Miami, FL, 33149-1098, USA.
[4]Departamento de Geomagnetismo y Exploración, Instituto de Geofísica, Universidad Nacional Autónoma de México, Ciudad Universitaria, México CDMX, 04510, México.
[5]NASA Goddard Space Flight Center, Greenbelt, MD, 20771, USA.
[*]Correspondence to: dario.e.solano@gmail.com, dsolano@unam.mx

This PDF file includes:

Supplementary Text S1
Figs. S1 to S10
Tables S1 to S4

Other Supplementary Materials for this manuscript include the following:

Data S1 to S6

Supplementary Text

S1. Reports of malfunctioning and damage to the Metro system relevant to the differential land subsidence analysis.

The following list of events summarises damage reports on the Metro system due to land subsidence from 2010 to 2022.

- In 2010, structural collapses, faults, cracks, deformation of the railways and apparent emersion were reported in Lines 4, 5, 9, A and B, as well as horizontal and vertical displacements of the railway's base that produced instability in the elevated sections, compromising structures in the section Puebla-Pantitlán, and the elevated aisles of stations Talisman, Bondojito, Consulado (Line 4) and south side of Pantitlán station[1]. The elevated section of Line B and 60% of Line 9 experienced apparent emersions with velocities from 3.6 to 4 cm/yr at the time that railways in lines 4 and 5 stand deformation that forced speed limit reductions of the trains[1].

- In March 2014, the Metro system suspended operations in 11 of the 20 stations of line 12 (corresponding to the elevated segment) for a period of 20 months[2]. This occurred only 17 months after the line's inauguration in October 2012. Repairs were needed after reported railway deformation and excessive friction between the railways and the train steel wheels, allegedly due to errors in planning, design, construction and operation, and lack of maintenance of the also called golden line[3–6]. Such service interruption increased the commuting time for users by up to 40 minutes[7].

- In August 2014, the National Labor Union of workers of the STC reported the presence of subsidence-related points of interest along line A, that proliferate in the segment from Tepalcates to La Paz[8].

- In May 2015, a crash occurred in Station Oceania (Line 5). The crash was attributed to human error when activating the braking system during a heavy storm, leaving 12 people injured[9]. This took place in a surface segment of the Metro where the slope has increased due to the compaction of the soft soils in the surroundings of Peñón de los Baños. Consequently, in July 2015 the Metro announced the construction of a tunnel to go through the Peñón de Los Baños, in order to avoid the deforming area[10]. The original slope of 3.5% had changed to 7.2% from 1981 to 2015 due to regional subsidence, well beyond the maximum slope of 4% established by the local regulations[10,11].

- In June 2015, the Metro system announced a 10-week service suspension on 5 of the 10 Line A stations. This happened in the sector from Peñón Viejo to La Paz, during operations of re-levelling of the railroads affected by differential subsidence[12]. It was the fifth time in 24 years that the line was repaired due to differential subsidence in the area. After the repairs, trains were announced to reach speeds of 90 km/hr instead of 45 km/hr before repairs[13]. Official sources report damage to infrastructure, including railways and foundations due to differential movements of the ground, producing longer commute times, risk to passengers, accessibility issues, and loss of comfort for riders. The foundation and walls are permanently compromised as part of the deformation process, as well as corrosion in the metallic parts of the structure, especially between Guelatao and La Paz Stations, where re-levelling is performed periodically[14].

- In July 2015, riders reported subsidence-induced ramps in the surface of up to 50 cm in station Pantitlán (Line 9)[15] and Oceania[16], as well as differential movements that have affected the railways, producing waves and changes in the slope, and consequently affecting the speed of the trains[15].

- In October 2015, the system announced major repairs in lines 1, 2 and 3, including re-levelling of the railways[17].

- In March 2016, a crash was reported in Line 5. Two passenger cars derailed after a mechanical malfunction while a train was arriving at station Politécnico, leaving only material damages[18]. However, this accident was not related, apparently, to ground deformation.

- Several railway-flood events led to service interruptions of the metro Line A after heavy precipitation (e.g. 13 June 2013[19], 24 Sept 2016[20], 14 June 2018[21], 13 May 2021[22]) between Peñón Viejo and La Paz Stations. Additionally, repeated re-levelling operations along LA have increased the elevation difference between the railways and the streets parallel to them as shown in Fig. 1D, and therefore, water flow towards the railways is facilitated in those railway sections standing at a lower elevation than the adjacent streets[21].

- In September 2018, the city's government releases a document describing and providing solutions to the main problems affecting the Metro system, among which the subsidence affecting lines 3, 5, and 9 is mentioned[23].

- In February 2019, the speed of trains travelling along line B is reduced, reportedly due to differential displacements affecting the line's electrical supply lines[24].

- In July 2019, street-level stations Indios Verdes, Deportivo 18 de Marzo, Potrero, and La Raza were closed for two weekends due to subsidence producing unevenness in the railways[25].
- In March 2021, line A's elevated stations Ciudad Deportiva, Puebla, and Pantitlán were closed to correct unevenness along the railways due to differential subsidence of the supporting columns[26].
- On May 3rd, 2021, an overpass segment of Line 12 collapsed onto the road as a train was travelling over it, very close to Olivos Station[27]. The collapse left at least 79 people injured and 24 dead[28]. Following the collapse, the government promised a forensic analysis[28], for which the company DNV was hired, delivering two preliminary reports on the apparent reasons leading to the accident[29,30]. Such analysis pointed out deficiencies in the design, construction, and maintenance of the collapsed structure, and indicated that differential displacements between columns could have played a role in the collapse, even when no measurements of such were available to be included in the analysis.
- During May 2021, and following the Olivos station overpass collapse, the structural health of several other structures raised concerns. For instance, the trains travelling along line 9's elevated overpasses, from Jamaica to Pantitlán stations, reduced their speed from 75 to 35 km-hr[31]. Several other areas, such as Pantitlán, Oceanía, Romero Rubio, San Lázaro and line A raised concerns as well[32].
- On May 11, 2022, Mexico City's government disallows the third and final report developed by the company DNV about the root causes leading to the May 3rd Olivos accident, despite the two preliminary reports previously delivered to the government[33,34]. The government deemed such a final report inadequate due to three main reasons[33]:
  - The analysis indicated a lack of routinary inspections, even though results from 2019 and 2020 inspections were provided to DNV.
  - The third report indicates that pre-collapse damage is identifiable from Google Street View imagery. However, the second report indicates that structural damage was not recognizable from in-situ pre-collapse imagery and visual inspections.
  - Limit states indicated in the third report were not evaluated considering the tolerable thresholds indicated in the local construction codes.
- In August 2022, damage is reported at San Lázaro station (elevated segment along Line B)[35], which led to prompt action from the government a few days later to ensure the stability of the line[36].

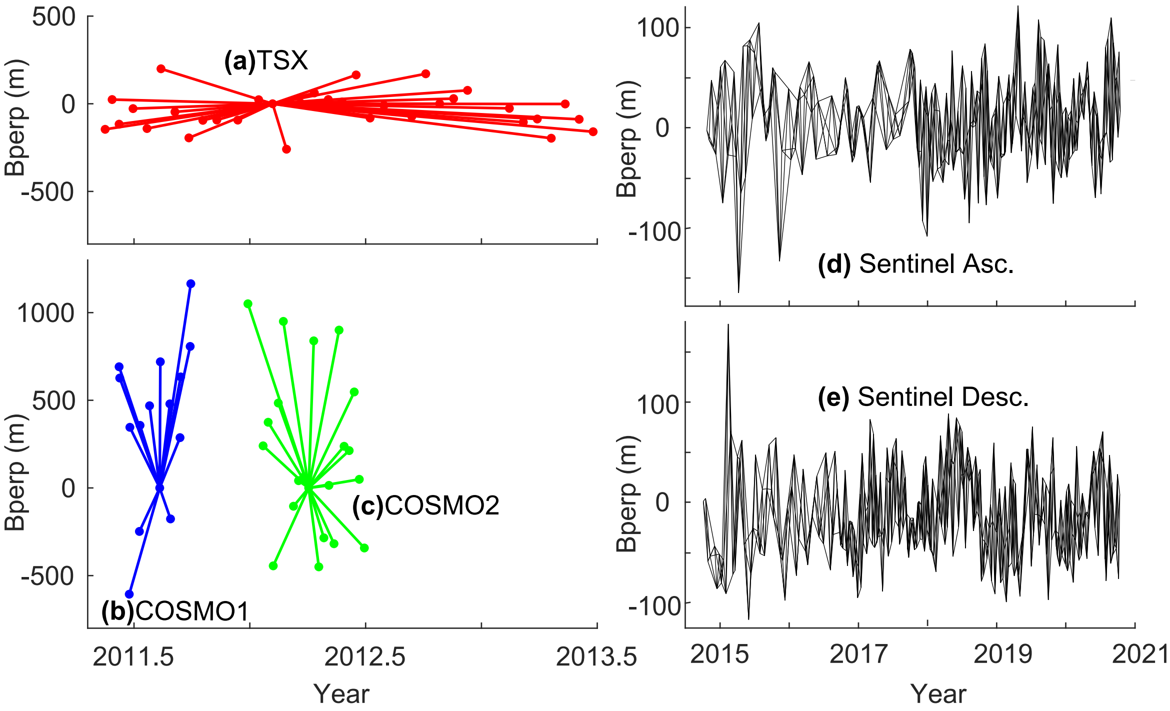

Figure S1. Temporal and perpendicular-baseline (Bperp) networks for each dataset. (a) TSX (b) COSMO1 and (c) COSMO2 single-master networks, where each line represents an interferogram constructed with the information in Supplementary Table S2 and S3. Perpendicular baselines in COSMO1 and COSMO2 datasets are clearly larger than in the TSX one. However, the TSX dataset includes interferograms with longer temporal baselines due to the longer temporal coverage. (d) Sentinel Ascending and (e) Sentinel descending multi-master networks. Notice the larger temporal coverage of both Sentinel datasets, as compared to (a-c).

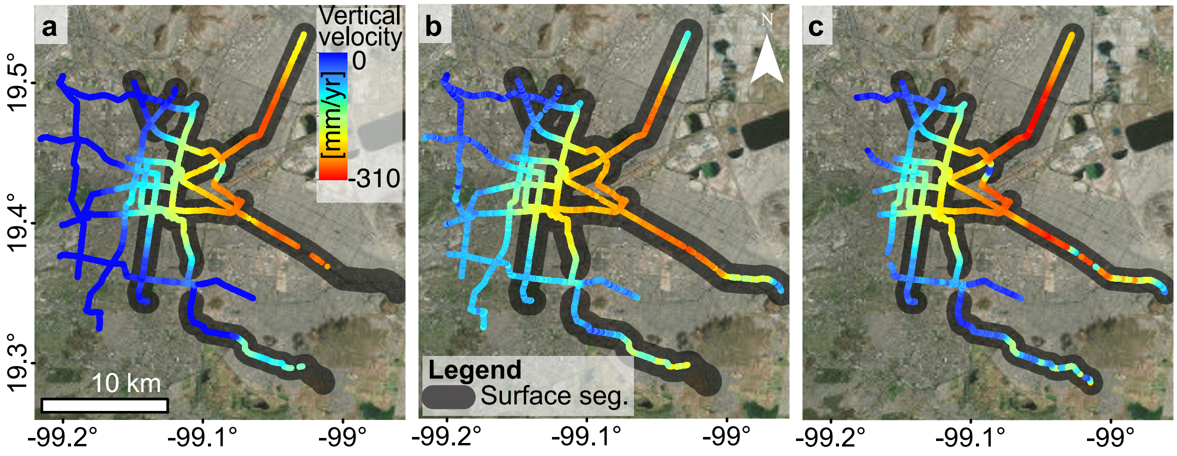

Figure S2. Vertical velocities along the Metro lines from the three X-band datasets. (a) TSX, (b) COSMO1, and (c) COSMO2 vertical velocities along the trace of both surface (the focus of this study, indicated by gray patches) and underground Metro line segments.

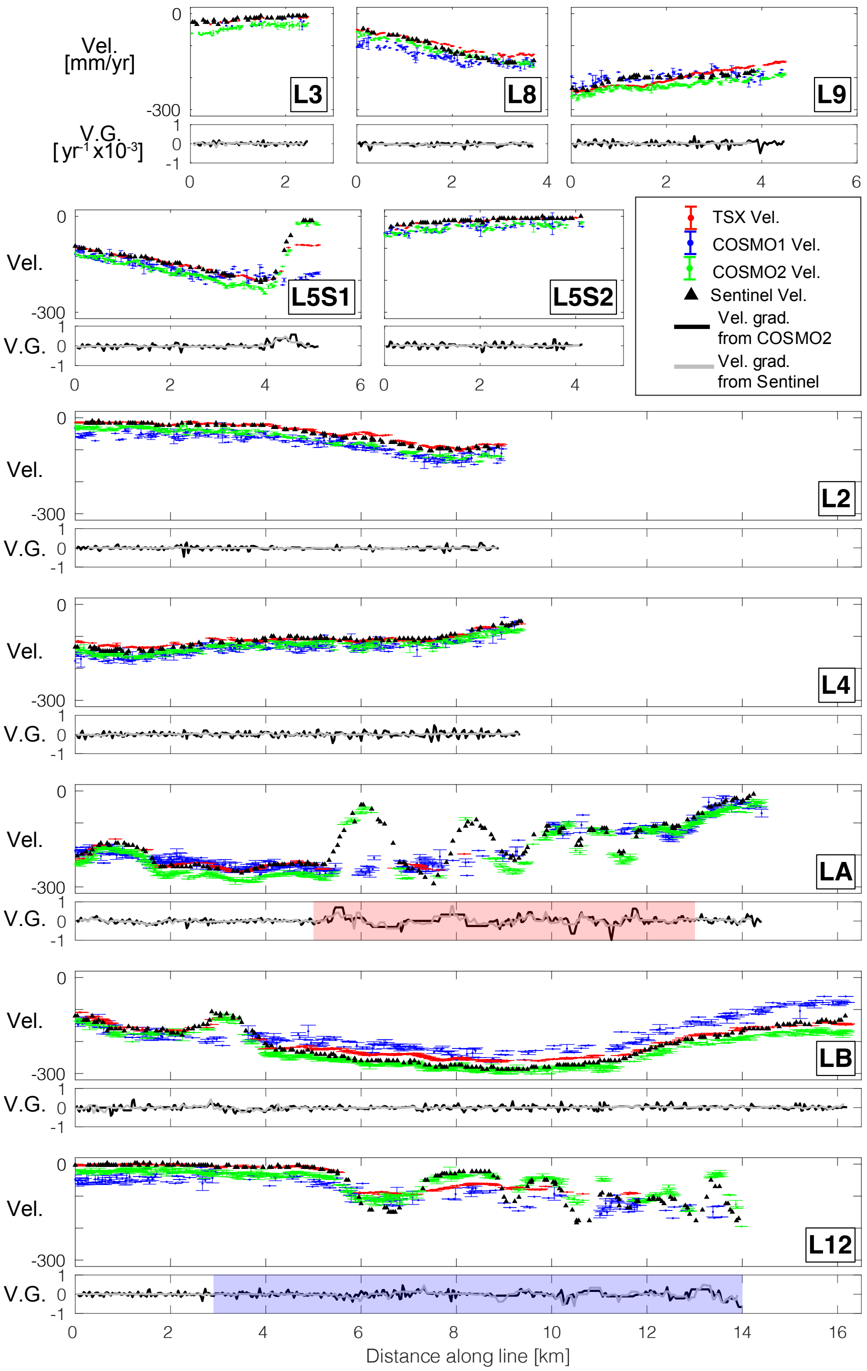

Figure S3. Vertical velocity profiles and calculated velocity gradients along surface segments of the Metro system. Labels correspond to the location of surface segments indicated in Fig. 2C. The presented velocities represent 30x10 rectangles along each metro line. In the case of COSMO1, COSMO2 and TSX results, the velocity corresponds to the average calculated from all available samples within such rectangles and is presented along with an error bar. In the case of Sentinel results, the displayed velocity corresponds to the sample closest to the centre of each rectangle. Below each velocity profile, the calculated velocity gradient from COSMO2 and Sentinel vertical velocities are presented. Red and blue patches correspond segments with subsidence-related damage reports along Line A and Line 12, respectively (compare to damage reports indicated in Fig. 2C). Vel= Vertical velocity, and V.G= Velocity gradient.

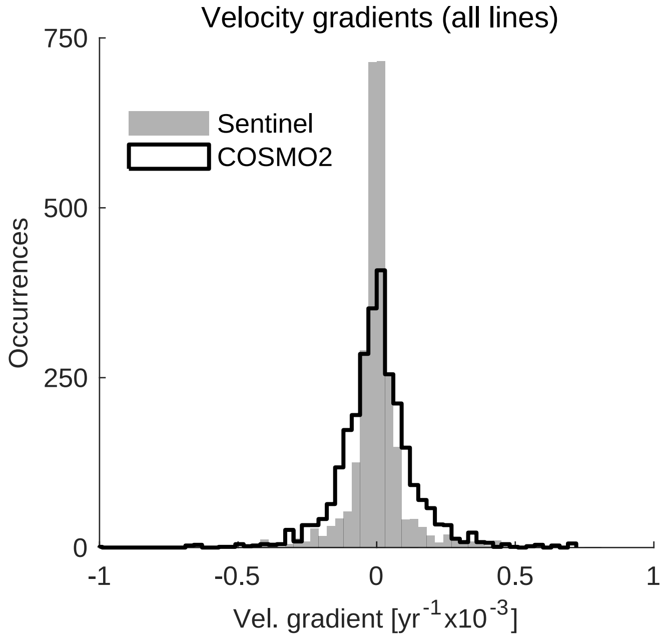

Figure S4. Comparison of velocity gradient results. Histograms of the results from calculating the velocity gradient from Sentinel data and from COSMO 2 results. These data for each histogram include the gradients shown in Supplementary Fig. S3 for all lines.

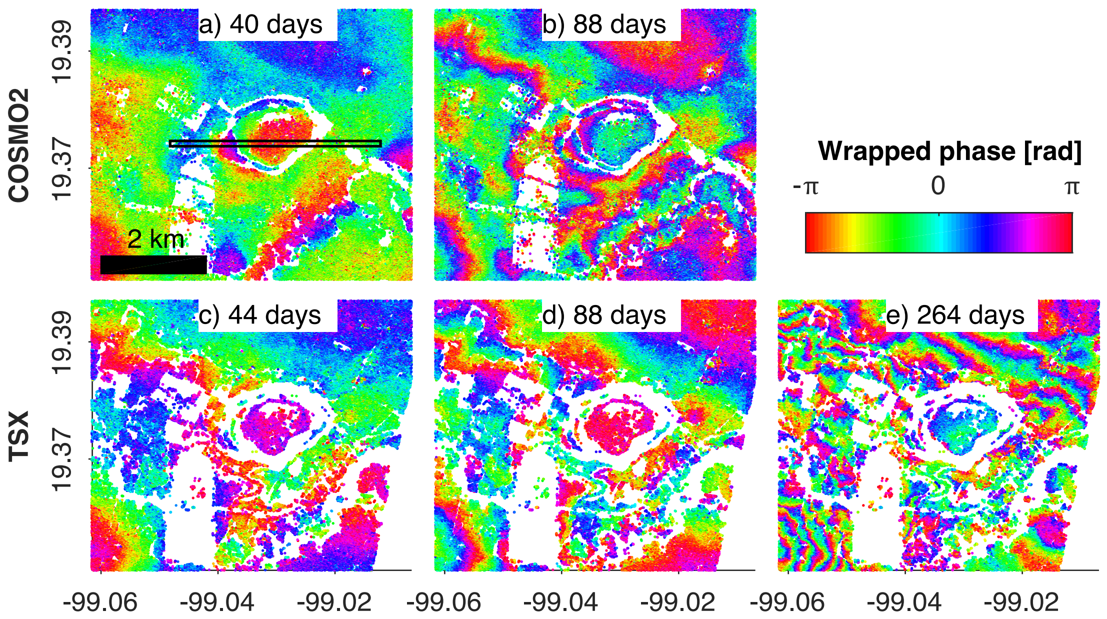

Figure S5. Examples of wrapped interferograms around Peñón Viejo. COSMO2 interferograms formed by the dates 2012/04/03-2012/05/13 (a) and 2012/04/03-2012/06/30 (b), and TSX interferograms formed by the dates 2012/02/06-2012/03/21 (c), 2012/02/06-2012/05/04 (d), and 2012/02/06-2012/10/27 (e). Sampling points correspond to the location of PSs selected in the StaMPS processing of each dataset. Notice that TSX interferograms have larger areas with poor PS density, as compared to COSMO2 results. The black polygon delimits the area used to generate the profiles in Supplementary Fig. S6.

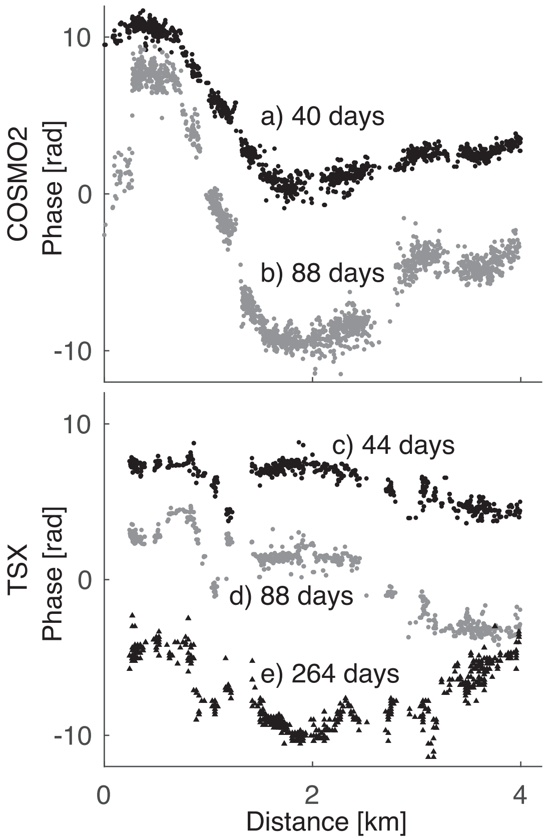

Figure S6. Results of performing 1D phase unwrapping to Supplementary Fig. S4 data along the transect shown in Fig. S5A. (a, b) 1D phase unwrapping results from COSMO2 40 and 80-day interferograms. (c-e) 1D phase unwrapping results from TSX 44, 80 and 264-day interferograms. Notice that fewer sample points (i.e. fewer available PS) and larger gaps in the TSX dataset lead to unwrapping errors even for a 44-day interferogram (compare (a) and (c)). The profiles are vertically shifted for clarity.

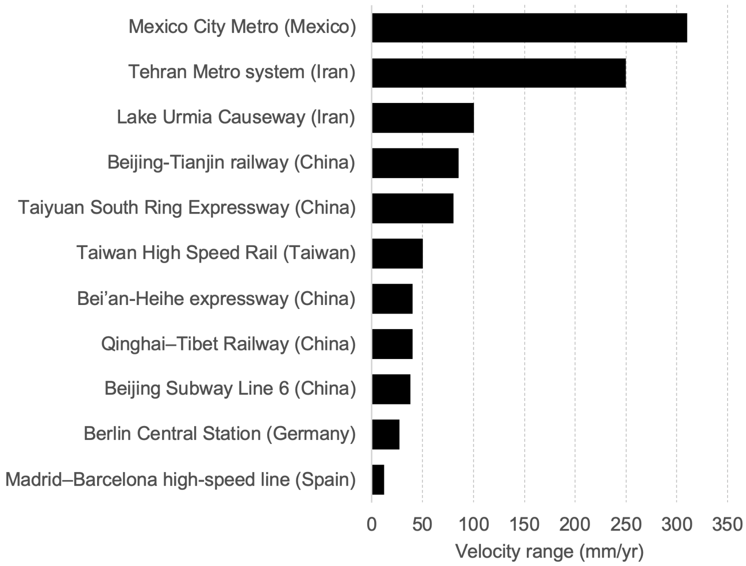

Figure S7. Range of subsidence velocities along infrastructure features worldwide. Maximum velocity range reported from the literature as compiled in Supplementary Table S4.

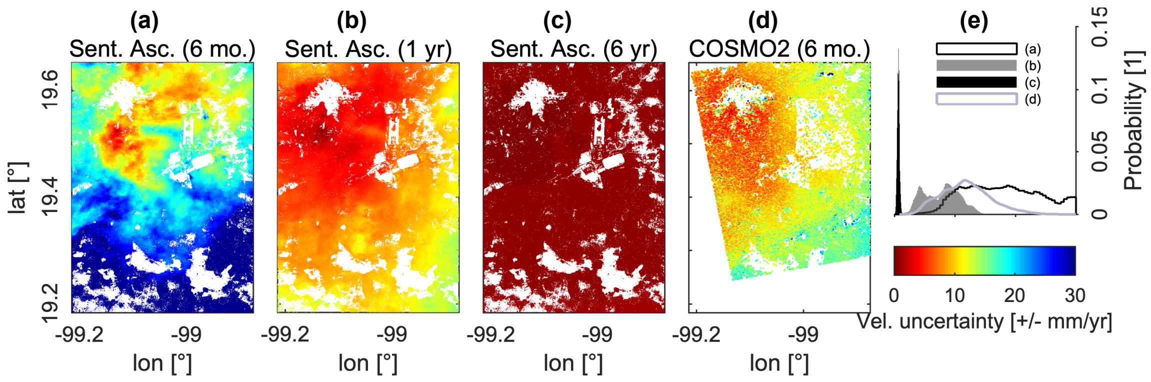

Figure S8. Comparison of velocity uncertainties obtained from Sentinel data in Ascending orbit as compared to COSMO2 dataset.(a) Uncertainties resulting from inverting only the SAR scenes acquired in the first six months of data from the start date of Oct 2014. (b) Same as (a) but for one-year worth of data. (c) Uncertainties calculated from inverting the whole dataset, which corresponds to six years of data. (d) Uncertainties corresponding to the COSMO2 dataset, which was calculated using PSI. (e) Histograms showing the distribution of the uncertainties shown in (a)-(d).

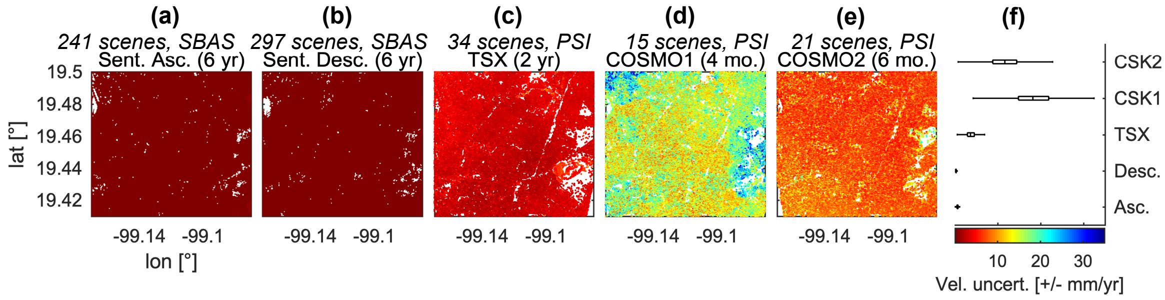

Figure S9. Comparison of velocity uncertainties obtained from all the datasets processed in this work. (a) and (b) show the uncertainties from the two six-year-long C-band datasets processed using SBAS. (c)-(e) show the results from processing the three X-band datasets processed using PSI. (f) Boxplots showing the distribution of the data shown in (a)-(e) for comparison.

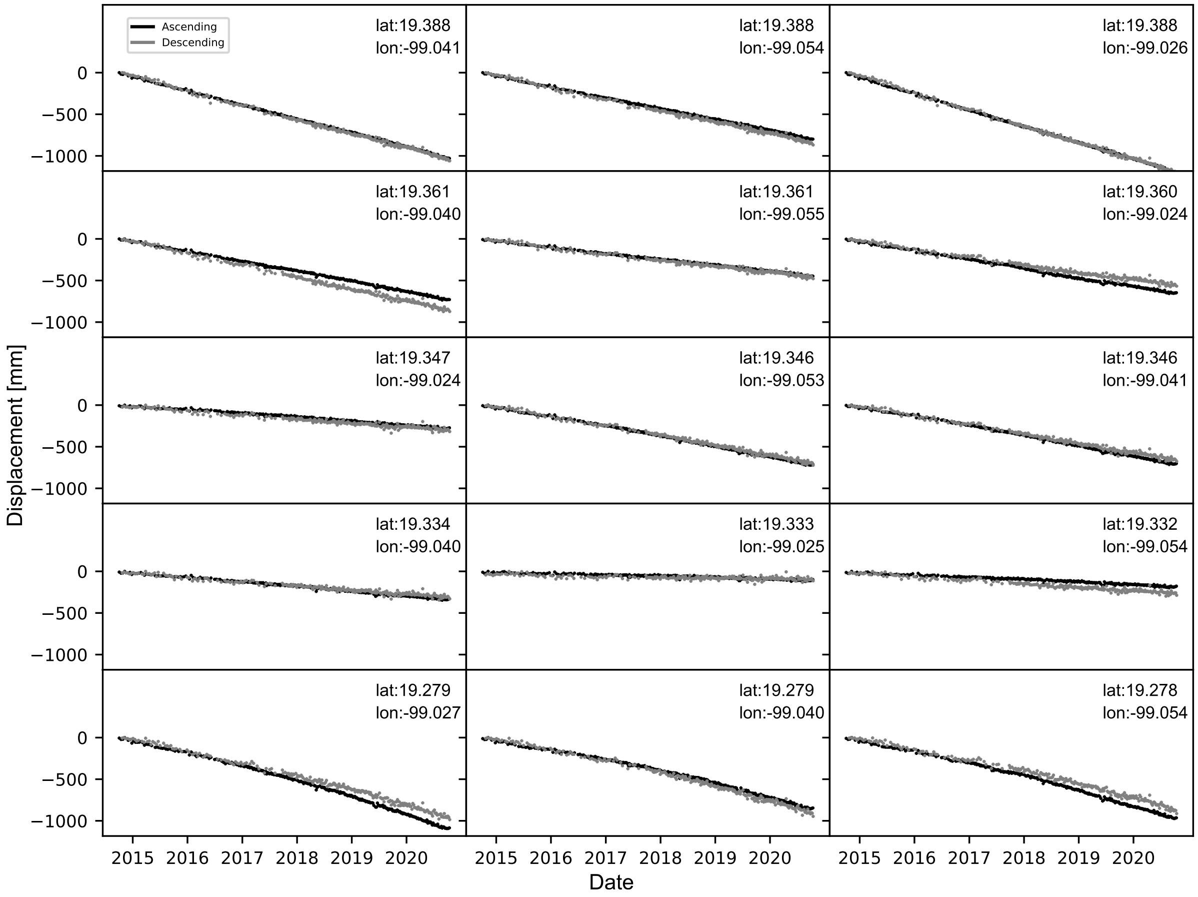

Figure S10. Examples of time series from Sentinel results in both Ascending and Descending orbits. The coordinates of the displayed data are shown in the upper right corner of each pane.

Table S1. Summary of reports of damage or malfunctioning on the Metro system (2010-2022).

Metro line or station	Damage				Malfunctioning				Land subsidence is a reported factor	Reference
	Collapses, faults and cracks	Apparent uplift	Railways deformation	Slope changes	Speed reduction of the trains	Crash/accident	Service interruption	Railway flooding		
1			X	X					X	14, 17
4	X	X	X		X				X	1
5	X	X	X	X	X	X			X	1, 9–11, 18
A	X	X	X	X	X		X	X	X	1, 8, 12–14, 19–21, 37
B	X	X	X						X	1
12			X		X	X	X		X	2, 4–7, 27, 28
Oceanía		X	X	X	X	X			X	1, 16
Pantitlán	X	X							X	1, 15, 16
Olivos	X					X			X	27, 28

Table S2. X-band SAR scenes from the three X-band datasets used in this study.

TSX		COSMO1			COSMO2		
Date	Bperp.	Date	Platform	Bperp.	Date	Platform	Bperp.
18-May-2011	-145.8	9-Jun-2011	CSKS2	691.2	29-Dic-2011	CSKS2	1050.4
29-May-2011	24	10-Jun-2011	CSKS3	626.9	22-Jan-2012	CSKS1	240.2
9-Jun-2011	-116.4	25-Jun-2011	CSKS2	-606.2	30-Jan-2012	CSKS2	374.6
1-Jul-2011	-27.6	26-Jun-2011	CSKS3	345.9	7-Feb-2012	CSKS1	-444.4
23-Jul-2011	-141.3	11-Jul-2011	CSKS2	-248.1	15-Feb-2012	CSKS2	484.3
14-Aug-2011	199.5	12-Jul-2011	CSKS3	357	23-Feb-2012	CSKS1	950
5-Sep-2011	-46.4	27-Jul-2011	CSKS2	468.8	10-Mar-2012	CSKS1	-104.4
27-Sep-2011	-194.6	12-Aug-2011	CSKS2	0	18-Mar-2012	CSKS2	41.5
19-Oct-2011	-94.5	13-Aug-2011	CSKS3	719.6	26-Mar-2012	CSKS1	61.8
10-Nov-2011	-93	28-Aug-2011	CSKS2	479.5	3-Apr-2012	CSKS2	0
13-Dec-2011	-93.2	29-Aug-2011	CSKS3	-176.6	11-Apr-2012	CSKS1	839
24-Dec-2011	-25.4	13-Sep-2011	CSKS2	286.9	19-Apr-2012	CSKS2	-450.1
15-Jan-2012	23.1	14-Sep-2011	CSKS3	633.2	27-Apr-2012	CSKS1	-284.6
6-Feb-2012	0	29-Sep-2011	CSKS2	807.1	5-May-2012	CSKS2	14.7
28-Feb-2012	-259.2	30-Sep-2011	CSKS3	1164.8	13-May-2012	CSKS1	-318.2
21-Mar-2012	4.3				21-May-2012	CSKS2	900.3
12-Apr-2012	61.8				29-May-2012	CSKS1	237.6
4-May-2012	24.3				6-Jun-2012	CSKS2	213
26-May-2012	10.6				14-Jun-2012	CSKS1	547.5
17-Jun-2012	164.4				22-Jun-2012	CSKS2	49.1
9-Jul-2012	-81.5				30-Jun-2012	CSKS1	-342.5
31-Jul-2012	-9.2						
13-Sep-2012	-72.5						
5-Oct-2012	171						
27-Oct-2012	0.5						
18-Nov-2012	29.4						
10-Dec-2012	76.5						
14-Feb-2013	-26.2						
8-Mar-2013	-105						
30-Mar-2013	-86.9						
21-Apr-2013	-196.6						
13-May-2013	-1.2						
4-Jun-2013	-88.6						
26-Jun-2013	-159.5						

Table S3. Characteristics of the five SAR datasets used in this study for InSAR processing.

Parameter	Dataset				
	TSX	COSMO1	COSMO2	Sentinel Asc.	Sentinel Desc.
Satellite(s)	TerraSAR-X	COSMO-SkyMed 2 and 3	COSMO-SkyMed 1 and 2	Sentinel-1A, B	Sentinel-1A, B
Average incidence angle [°]	30.4	26.7	38.8	43.97	43.99
Area covered [km2]	1035	1815	1681	1515	1515
Number of acquisitions	34	15	21	241	297
Acquisition mode	StripMap	HIMAGE	HIMAGE	IW	IW
Acquisition direction	Descending	Ascending	Ascending	Ascending	Descending
Start date	18-May-2011	9-Jun-2011	29-Dec-2011	23-Oct-2014	3-Oct-2014
End date	26-Jun-2013	30-Sep-2011	30-Jun-2012	15-Oct-2020	13-Oct-2020
Max. temporal baseline [days]	506	64	96	48	48
Max. perpendicular baseline [m]	259.2	1164.8	1050.4	162	178
Polarization	HH	HH	HH	VV	VV
Master date	20120206	20110812	20120403	Multiple	Multiple
Master scene absolute orbit	25770	19885	23366	2950	2665

Table S4. Non-comprehensive compilation of studies on infrastructure features affected by subsidence worldwide.

Feature observed and country	Reference	Observation period	Geodetic technique	Velocity					
				Direction	Max. in study areas (mm/yr)	Min. in study area (mm/yr)	Max. along structure (mm/yr)	Min. along structure (mm/yr)	Range along structure
Bei'an-Heihe expressway, China	38	2012	InSAR	LOS	N/A	N/A	0	-40	40
Beijing Subway Line 6, China	39	2010-2014	InSAR, levelling	LOS	20	-76	-5	-43	38
Beijing-Tianjin railway, China	40	2004-2010	InSAR	Vert.	20	-136	-6	-80	74
Beijing-Tianjin railway, China	41	2008-2009	InSAR	LOS*	0	19	0	-10	10
Beijing-Tianjin railway, China	42	2003-2004	InSAR	LOS*	5	-141	0	-83	83
Beijing-Tianjin railway, China	43	2003-2015	InSAR, leveling	Vert.	0	-115	0	-85	85
Berlin Central Station, Germany	44	2008-2013	InSAR photogrammetry	LOS	N/A	N/A	12	-15	27
Madrid–Barcelona high-speed line, Spain	45	2003-2010	InSAR	LOS	N/A	N/A	2.1	-9.7	11.8
Mexico City Metro, Mexico	This work	2011-2013	InSAR	Vert.	20	-406	0	-310	310
Qinghai–Tibet Railway, China	46	2007-2010	InSAR	LOS	20	-20	N/A	N/A	N/A
Qinghai–Tibet Railway, China	47	2003-2012	InSAR	LOS	>20	<-20	20	-20	40
Shanghai Metro and highways, China	48	2009-2010	InSAR	LOS*	5	-25	N/A	N/A	N/A
Taiwan High Speed Rail, Taiwan	49	2003-2006	Leveling, GPS, monitoring wells	Vert.	N/A	N/A	0	-50	50
Taiwan High Speed Rail, Taiwan	50	2006-2007	Leveling, GPS, monitoring wells, InSAR	Vert.	0	-122	N/A	-60	N/A
Taiyuan South Ring Expressway, China	51	2006-2009	InSAR	Vert.*	-10	-110	-10	-90	80
Tehran Metro System, Iran	52	2003-2017	InSAR leveling	Vert.	0	<-250	0	-250	250
Lake Urmia Causeway, Iran	53	2004-2017	InSAR	Vert.	0	-100	0	-100	100

N/A: Not Available from the reference.
*Measurement direction was not specified in the reference, but inferred from the study's context

Data S1. (S1_Date_SAR_scenes_ascending.txt).
Acquisition dates of the Sentinel SAR ascending track scenes used to produce the velocity map shown in Fig. 3D.

Data S2. (S2_Date_SAR_scenes_descending.txt).
Acquisition dates of the Sentinel SAR descending track scenes used to produce the velocity map shown in Fig. 3E.

Data S3. (S3_street_level_gradients.kmz).
Kml file containing velocity gradients along street-level railways of the Metro system. When opened in Google Earth, displayed colours coincide with Fig. 7a colour scale.

Data S4. (S4_elevated_gradients.kmz).
Kml file containing velocity gradients along elevated segments of the Metro system. When opened in Google Earth, displayed colours coincide with Fig. 8a colour scale.

Data S5. (S5_Santa_catarina_CKS1.kmz).
Kml file containing a subset of COSMO1 velocity results from Fig. 3B over Sierra de Santa Catarina.

Data S6. (S6_Santa_catarina_CKS2.kmz).
Kml file containing a subset of COSMO2 velocity results from Fig. 3C over Sierra de Santa Catarina.

References

1. Llanos, R. Cinco líneas del Metro, afectadas por hundimientos diferenciales: Bojórquez (2010).
2. Martinez-Brooks, D. Línea 12 del metro reabre todas sus estaciones tras 20 meses (2015).
3. Notimex. Sindicato del Metro advierte de posible descarrilamiento de Línea 12 (2015).
4. Milenio Diario. Las fechas clave en el conflicto de la Línea 12 del Metro (2014).
5. Secretaría de Obras y Servicios. Rehabilitación Línea 12 (2015).
6. SYSTRA. Diagnóstico de la Línea 12. Informe final. Tech. Rep., " (2014).
7. Ascension, A. El 'recorte' en la línea 12 del metro 'cobra pasaje' a los usuarios (2014).
8. Navarro, I. Línea A: 58 puntos con riesgos de hundimiento (2014).
9. Excelsior. Deja 12 lesionados choque de trenes en estación Oceanía (2015).
10. Metro. Proyecto ejecutivo civil, electromecánico y electrónico para la reconstrucción derivada de la modificación del perfil de vías del tramo Oceanía – Terminal Aérea de la Línea 5 del Sistema de Transporte Colectivo. (2015).
11. Metro. Dictamen técnico Oceanía (2015).
12. Sistema de Transporte Colectivo. Rehabilitación de la Línea "A" por quinta ocasión en 24 años, en uno de los suelos más dificiles (2015).
13. Pérez, L. Línea A del Metro alcanzará 90 km por hora (2015).
14. Sistema de Transporte Colectivo. Fideicomiso maestro del Metro: once compromisos asumidos por el Sistema de Transporte Colectivo. Tech. Rep., " (2014).
15. Hernández, A. Crecen grietas en paredes y pisos del Metro Pantitlán (2015).
16. Hernández, E. & Delgado, D. Denuncian hundimientos en estaciones del Metro.
17. La Silla Rota. Anuncia Gaviño reparación en líneas 1, 2 y 3 del Metro (2015).
18. El Economista. Descarrila vagón del Metro en la estación Politécnico (2016).
19. Pasos, F. Inundación en Línea A del Metro alcanza más de un metro de altura (2013).
20. Obras web. Construirán colector pluvial para evitar inundaciones en la Línea A (2016).
21. La Silla Rota. Metro culpa a la basura de inundación en Línea A (2018).
22. Redacción. Línea A reanuda servicio tras inundación en vías por fuertes lluvias (2021).
23. Sistema de Transporte Colectivo. Plan Maestro del Metro 2018 – 2030. Tech. Rep., Gobierno de la Ciudad de México, Mexico City, Mexico (2018).
24. Peralta, C. Línea por Línea: las fallas que afectan al Metro (2019). Section: Noticias.
25. Martínez, D. Metro cerrará cuatro estaciones de la línea 3 (2019).
26. Estrada, R. ¡Ojo! Cerrarán 3 estaciones de la Línea 9 del Metro; habrá transporte gratuito (2021).
27. Jefatura de Gobierno de la Ciudad de México. Atiende Gobierno de la Ciudad de México y Gobierno de México incidente en L-12 del Metro (2021).
28. Jefatura de Gobierno de la Ciudad de México. Informa Gobierno capitalino que se realizará dictamen de todos los tramos elevados de la Línea 12 del Metro (2021).
29. DNV. Dictamen técnico del incidente ocurrido en la línea 12 en el tramo elevado entre las estaciones Olivos y Tezonco, entre las columnas 12 y 13, y análisis de causa-raíz. Dictamen preliminar fase I. Tech. Rep., DNV Energy Systems Mexico S. de R.L. de C.V., Mexico City (2021).
30. DNV. Dictamen técnico del incidente ocurrido en la línea 12 en el tramo elevado entre las estaciones Olivos y Tezonco, entre las columnas 12 y 13, y análisis de causa-raíz. Dictamen final fase II. Tech. Rep., DNV Energy Systems Mexico S. de R.L. de C.V., Mexico City (2021).
31. Camarillo, M. Con apoyo de colegios de ingenieros se revisarán estructuras elevadas del Metro (2021). Section: Metrópoli.
32. Zamarrón, I. CDMX no quiere otra sorpresa: anuncia revisión de tramos elevados de líneas del Metro (2021).

33. Informe de las inconsistencias y contradicciones técnicas y científicas del reporte del análisis de resultados de causa-raíz elaborado por DNV Fase III NO ACEPTADO POR LA SECRETARÍA DE GESTIÓN INTEGRAL DE RIESGOS Y PROTECCIÓN CIVIL (2022).

34. Pérez, M. Ve CDMX contradicciones en informe final de DNV (2022).

35. Martínez, O. Reportan hundimiento dentro de estación San Lázaro del Metro de CDMX (2022).

36. González, R. Puente en la línea B del Metro no es de peligro (2022).

37. Ciudadanos en Red. Cerrarán cinco estaciones del Metro (2015).

38. Shi, X. *et al.* Expressway deformation mapping using high-resolution TerraSAR-X images. *Remote. Sens. Lett.* **5**, 194–203, DOI: 10.1080/2150704X.2014.891774 (2014).

39. Chen, W.-F. *et al.* Spatiotemporal evolution of land subsidence around a subway using InSAR time-series and the entropy method. *GIScience & Remote. Sens.* **00**, 1–17, DOI: 10.1080/15481603.2016.1257297 (2016).

40. GuangYao, D. *et al.* Monitoring and analysis of land subsidence along Beijing-Tianjin inter-city railway. *J. Indian Soc. Remote. Sens.* **44**, 915–931, DOI: 10.1007/s12524-016-0556-7 (2016).

41. Zhang, H., Tao, L., Wang, C. & Tang, Y. X. Ground deformation detection along Beijing-Tianjin intercity railway using advanced network multi-baseline DInSAR. In *Procedings of the 2010 International Conference on Wavelet Analysis and Pattern Recognition, ICWAPR 2010*, July, 222–226, DOI: 10.1109/ICWAPR.2010.5576330 (2010).

42. Ge, D., Wang, Y., Xia, Y., Guo, X. & Wang, Y. Land subsidence investigation along railway using permanent scatterers SAR interferometry. *Int. Geosci. Remote. Sens. Symp. (IGARSS)* **2**, 1235–1238, DOI: 10.1109/IGARSS.2008.4779225 (2008).

43. Chen, B. *et al.* Investigating land subsidence and its causes along Beijing high-speed railway using multi-platform InSAR and a maximum entropy model. *Int. J. Appl. Earth Obs. Geoinformation* **96**, 102284, DOI: 10.1016/j.jag.2020.102284 (2021).

44. Wang, Y., Zhu, X. X., Zeisl, B. & Pollefeys, M. Fusing Meter-Resolution 4-D InSAR Point Clouds and Optical Images for Semantic Urban Infrastructure Monitoring. *IEEE Transactions on Geosci. Remote. Sens.* **55**, 14–26, DOI: 10.1109/TGRS.2016.2554563 (2016).

45. Galve, J. P., Castañeda, C. & Gutiérrez, F. Railway deformation detected by DInSAR over active sinkholes in the Ebro Valley evaporite karst, Spain. *Nat. Hazards Earth Syst. Sci.* **15**, 2439–2448, DOI: 10.5194/nhess-15-2439-2015 (2015).

46. Chen, F., Lin, H., Li, Z., Chen, Q. & Zhou, J. Interaction between permafrost and infrastructure along the Qinghai-Tibet Railway detected via jointly analysis of C- and L-band small baseline SAR interferometry. *Remote. Sens. Environ.* **123**, 532–540, DOI: 10.1016/j.rse.2012.04.020 (2012).

47. Zhang, Q., Li, Y., Zhang, J. & Luo, Y. InSAR technique applied to the monitoring of the qinghai-tibet railway. *Nat. Hazards Earth Syst. Sci.* **19**, 2229–2240, DOI: 10.5194/nhess-19-2229-2019 (2019).

48. Perissin, D., Wang, Z. & Lin, H. Shanghai subway tunnels and highways monitoring through Cosmo-SkyMed Persistent Scatterers. *ISPRS J. Photogramm. Remote. Sens.* **73**, 58–67, DOI: 10.1016/j.isprsjprs.2012.07.002 (2012).

49. Hwang, C., Hung, W. C. & Liu, C. H. Results of geodetic and geotechnical monitoring of subsidence for Taiwan High Speed Rail operation. *Nat. Hazards* **47**, 1–16, DOI: 10.1007/s11069-007-9211-5 (2008).

50. Hung, W. C. *et al.* Monitoring severe aquifer-system compaction and land subsidence in Taiwan using multiple sensors: Yunlin, the southern Choushui river Alluvial fan. *Environ. Earth Sci.* **59**, 1535–1548, DOI: 10.1007/s12665-009-0139-9 (2009).

51. Wu, H., Zhang, Y., Zhang, J. & Chen, X. Mapping deformation of man-made linear features using DInSAR technique. In *ISPRS TC VII Symposium – 100 Years ISPRS*, vol. XXXVIII, 293–297 (2010).

52. Haghshenas Haghighi, M. & Motagh, M. Ground surface response to continuous compaction of aquifer system in Tehran, Iran: Results from a long-term multi-sensor InSAR analysis. *Remote. Sens. Environ.* **221**, 534–550, DOI: 10.1016/j.rse.2018.11.003 (2019).

53. Karimzadeh, S., Matsuoka, M. & Ogushi, F. Spatiotemporal deformation patterns of the Lake Urmia Causeway as characterized by multisensor InSAR analysis. *Sci. Reports* **8**, 1–10, DOI: 10.1038/s41598-018-23650-6 (2018).
